# Ibuprofen prevents progression of ataxia telangiectasia symptoms in ATM-deficient mice

**DOI:** 10.1186/s12974-018-1338-7

**Published:** 2018-11-06

**Authors:** Chin Wai Hui, Xuan Song, Fulin Ma, Xuting Shen, Karl Herrup

**Affiliations:** 1 Division of Life Science and State Key Laboratory of Molecular Neurobiology, Hong Kong University of Science and Technology, Clear Water Bay, Kowloon, Hong Kong; 2 Present address: School of Biomedical Sciences, The University of Hong Kong, Pokfulam, Hong Kong

**Keywords:** Ataxia telangiectasia, Ibuprofen, Anti-inflammatory, Microglia, Purkinje cell

## Abstract

**Background:**

Inflammation plays a critical role in accelerating the progression of neurodegenerative diseases, such as Alzheimer’s disease (AD) and ataxia telangiectasia (A-T). In A-T mouse models, LPS-induced neuroinflammation advances the degenerative changes found in cerebellar Purkinje neurons both in vivo and in vitro. In the current study, we ask whether ibuprofen, a non-steroidal anti-inflammatory drug (NSAID), can have the opposite effect and delay the symptoms of the disease.

**Methods:**

We tested the beneficial effects of ibuprofen in both in vitro and in vivo models. Conditioned medium from LPS stimulated primary microglia (LM) applied to cultures of dissociated cortical neurons leads to numerous degenerative changes. Pretreatment of the neurons with ibuprofen, however, blocked this damage. Systemic injection of LPS into either adult wild-type or adult *Atm*^*−/−*^ mice produced an immune challenge that triggered profound behavioral, biochemical, and histological effects. We used a 2-week ibuprofen pretreatment regimen to investigate whether these LPS effects could be blocked. We also treated young presymptomatic *Atm*^*−/−*^ mice to determine if ibuprofen could delay the appearance of symptoms.

**Results:**

Adding ibuprofen directly to neuronal cultures significantly reduced LM-induced degeneration. Curiously, adding ibuprofen to the microglia cultures before the LPS challenge had little effect, thus implying a direct effect of the NSAID on the neuronal cultures. In vivo administration of ibuprofen to *Atm*^*−/−*^ animals before a systemic LPS immune challenge suppressed cytological damage. The ibuprofen effects were widespread as microglial activation, p38 phosphorylation, DNA damage, and neuronal cell cycle reentry were all reduced. Unfortunately, ibuprofen only slightly improved the LPS-induced behavioral deficits. Yet, while the behavioral symptoms could not be reversed once they were established in adult *Atm*^*−/−*^ animals, administration of ibuprofen to young mutant pups prevented their symptoms from appearing.

**Conclusion:**

Inflammatory processes impact the normal progression of A-T implying that modulation of the immune system can have therapeutic benefit for both the behavioral and cellular symptoms of this neurodegenerative disease.

**Electronic supplementary material:**

The online version of this article (10.1186/s12974-018-1338-7) contains supplementary material, which is available to authorized users.

## Background

Ataxia telangiectasia (A-T) is a neurodegenerative disease of childhood with a prevalence between 1 in 40,000 and 1 in 100,000 people worldwide. It results from the mutation of a single gene, A-T mutated (ATM), whose gene product is a large kinase of the PI3K family. A-T symptoms include a progressive neuronal loss, ataxia, cancer susceptibility, hypersensitivity to ionizing radiation, immunodeficiency, and sterility [1–3]. The particular type of immunodeficiency found in A-T is life-threatening for nearly one third of A-T patients because of the resulting increase in susceptibility to infections, particularly of the lung [4]. In the context of a neurodegenerative disease, the co-occurrence of these immune problems is noteworthy since the immune and nervous system maintain extensive communication through the entire lifespan [5, 6]. The development of the central nervous system (CNS) can be significantly altered by immune challenges, and in the adult, unchecked inflammatory signals and the resulting cytokine imbalances usually lead to fatigue, impaired cognition, as well as slower healing and recovery after nerve injury [7–12]. Chronic inflammation is found in many age-related disorders, where it raises the susceptibility to cardiovascular difficulties, asthma, and cancer [13]. Many late-onset neurodegenerative diseases are also associated with a long-term inflammatory process, and in vitro studies suggest that this process involves the release of a large profile of pro-inflammatory cytokines [14, 15] and reactive oxygen species (ROS) [16, 17]. In A-T patients, sustained immune challenges, including bacterial infections and chronic inflammation, greatly contribute to the development of disease pathology [18, 19]. Furthermore, reports from clinical trials have shown that glucocorticoids, powerful anti-inflammatory hormones, are able to ameliorate the symptoms of A-T [20, 21]. The finding of peripheral immune deficiency in A-T patients is well established and highlights the contribution of the immune system to the symptoms of A-T. The contribution of the immune system, in particular, the microglia of the brain’s innate immune system, to the neurological and neuropathological abnormalities of A-T remains less certain.

Previous studies in experimental systems have shown that microglia have a significant impact on the process of neurodegeneration in A-T in both mouse and Drosophila models [22, 23]. Studies from our lab have found that systemic LPS administration triggers exaggerated neuronal damage in both short (1 week) [24] and long (1 month) [25] time frames. In this study, we investigated the effect of ibuprofen, a non-selective non-steroidal anti-inflammatory drug (NSAID). NSAIDs are regarded as non-selective COX inhibitors to reduce neuroinflammation, promote neuronal survival, and improve cognitive function in rodent models of different neurodegenerative conditions—Alzheimer’s disease (AD), traumatic brain injury, and exaggerated excitotoxicity [26–28]. Epidemiological studies suggest that they are protective in human AD [29] and are of significant benefit in mouse models of AD if they are administered before symptoms appear [29, 30]. Unfortunately, prospective AD clinical trials showed little efficacy in subjects with mild dementia, but two human studies have shown that ibuprofen is able to modify the progression of mild AD [31] and can directly block LPS-induced microglial activation and impairments of spatial working memory [32]. These human studies have been replicated in mouse models of AD [30, 33–35] with the exception of the 5XFAD mouse [36].

Here, we report that in vitro ibuprofen pretreatment offers significant protection. In vivo *Atm*^*−/−*^ animals pretreated with ibuprofen for 2 weeks are less vulnerable to LPS-induced motor dysfunction, have less Purkinje cell damage, and show reduced microglial activation. In the absence of an exogenous immune challenge, ibuprofen treatment of young presymptomatic *Atm*^*−/−*^ animals improved multiple classic histopathological features of the *Atm*^*−/−*^ brain. These data support a potential role for ibuprofen in preventing the development of neuropathological symptoms in A-T. While ibuprofen did not improve the motor performance of the *Atm*^*−/−*^ mice in the treatment regimen we used, it proved to be effective in preventing new pathological signs from developing. Taken together, our data suggest that administration of the NSAID ibuprofen can impact the progression of A-T at both the cellular and organismal level.

## Methods and materials

### Atm-deficient mice

A breeding colony of mice with a targeted disruption of the Atm gene *Atm*^*tm1Awb*^ [37] was obtained from The Jackson Laboratory (Bar Harbor, ME). Generation of mutants was achieved through the mating of heterozygous *Atm*^*+/−*^ males and *Atm*^*+/−*^ females. The mice were maintained on a 129/SvJ genetic background. Genotyping was performed on extracted tail DNA using PCR techniques that were described previously [37]. All animal experimental protocols were approved both by the Animal Ethics Committee at HKUST and their care that was in accord with the institutional and Hong Kong guidelines.

### Injections with lipopolysaccharide

Lipopolysaccharide (LPS, *Escherichia coli* serotype 055:B5) was purchased from Sigma-Aldrich (L2880, St. Louis, MO, USA), dissolved in distilled water, and stored at − 20 °C. Adult mice (3-month-old) of *Atm*^+/+^and *Atm*^*−/−*^ genotypes were given daily intraperitoneal injections of LPS (1 mg/kg for a period of 4 days) in keeping with our previous protocol [24]. A control group was treated on the same schedule with injection of filtered saline only. Mice were killed on the fifth day, 24 h after the last injection. Mice in both groups were monitored carefully for signs of sickness or distress during the entire period. Following sacrifice, the brains were dissected and the tissues prepared as described below.

### Ibuprofen oral administration

Commercial ibuprofen was purchased from CVS pharmacy and kept at room temperature. Two treatment groups were established. In the first group, the dose of ibuprofen used (62.5 mg/kg) [38] was orally administrated to P10 mice of *Atm*^*+/+*^and *Atm*^*−/−*^ genotypes once per day for 2 weeks to investigate whether ibuprofen could block the development of the A-T symptoms that normally appear during this early postnatal period. A control group of the same age was untreated. Mice were killed as above immediately after receiving the last oral suspension. A second group of 3-month-old adult animals (*Atm*^*+/+*^ and *Atm*^*−/−*^ genotypes) were administrated ibuprofen (0.5 mg/ml of drinking water, calculated based on the amount of water consumed by mice and administration dose as mentioned above) for 2 weeks to investigate whether ibuprofen could prevent the progression of A-T symptoms. Mice then received daily intraperitoneal injections of LPS (1 mg/kg) for 4 days with or without oral administration of ibuprofen (62.5 mg/kg). Mice were killed on the fifth day, 24 h after the last injection. During the treatment, mice were monitored carefully.

### Rotarod test

After treatment with LPS or/and ibuprofen, mice were subjected to rotarod testing without initial training to measure motor coordination but not motor learning. Mice were placed on the rotating rod for 1 min then tested for coordination by measuring the time they were able to remain on the rod as the rotation speed accelerated (4–40 rpm with an acceleration of 4 rpm/10s). The experimental groups were randomized in different positions while running on the rods. The rotarod software (ANY-maze Behavior Tracking Software; Stoelting Co., Wood Dale, IL) calculates the number of times the animal complete an entire rotation of 360° during the observation period.

### Open field test

The open field test is used to determine gross locomotor activity and exploration habits. Mice were introduced singly into a square arena (50 × 50 cm) bounded by tall walls with a defined (but invisible) center area of 25 × 25 cm. Mice were allowed to acclimate to the testing room overnight before training. Mice were placed in the center of the square arena and allowed to freely move for 10 min while being tracked by an automated tracking system (Stoelting ANY-maze, Wood Dale, U.S.A). After 4 to 5 days of treatment with LPS or/and ibuprofen administration, the mice were tested every 2 days. Mice were killed immediately after the third test on the fifth day (Fig. 3c). The experimental groups were randomized in different open field platforms.

### Tissue preparation and histology

Animals were deeply anesthetized with Avertin (0.02 cc/g body weight) and perfused with cold PBS for 3 min. After perfusion, the brain was dissected out and bisected along the midline. Half of the brain was stored at − 80 °C for future use. Half was fixed in 4% paraformaldehyde (PFA, Sigma-Aldrich) at 4 °C overnight. After washing twice with PBS, the brain was transferred to 30% sucrose solution and incubated at 4 °C overnight for cryoprotection. Brains were then embedded in OCT and frozen quickly in powdered dry ice. Ten micron cryostat sections were cut and allowed to air dry on pre-coated *SuperPlus* glass slides.

### Primary microglia cell culture and preparation of LPS conditioned medium

Primary microglia were isolated from C57BL/6J mice using our established protocol [39]. Briefly, a mixed glial cell population was obtained from P2–P5 pups and was cultured for 2 weeks in DMEM medium supplemented with 10% FBS and 1% pen/strep. Pure microglia were obtained by shaking at 37 °C for 4 h. LPS conditioned medium (LM) was prepared by incubating microglia cells at a density of 50,000 per well in a 24-well plate in Neurobasal medium containing 10 μg/ml LPS for 2 days. In another group, ibuprofen (200 μM) was added to the microglial cultures for 6 h and before treating them with 10 μg/ml LPS for 2 days. A control group was treated in Neurobasal medium without LPS (MM). After the two-day treatment, the medium was obtained and centrifuged to remove cells and debris, then used for neuronal treatment within 24 h. Microglial cells were washed by PBS once and lysed for further studies. THP-1 cells were cultured in RPMI 1640 medium with 10% FBS and 0.05 mM β-mercaptoethanol for routine passage. β-mercaptoethanol was removed from the medium when cells were treated with stimulus.

### Primary cortical neuronal culture

Embryonic cortical neurons were isolated by standard procedures [40]. All cultures were grown for a minimum of 13 days in vitro (DIV) before any treatment. Cultures were then pretreated with ibuprofen (80, 120, and 200 μM) or DMSO for 24 h after which MM or LM (12.5% volume/volume) was added for another 48 h. For histological studies, cells were washed with PBS and fixed in 4% PFA for 15 min. After rinsing in PBS, cells were stored in 0.1% PFA if longer-term storage was required.

### Antibodies for histological and Western studies

PCNA, p38, phospho-p38 (Thr180/Tyr182), ERK, phospho-ERK (Thr202/Tyr204), JNK, phospho-JNK (Thr183/Tyr185), NFκB p105/50, NFκB p65, phospho-NFκB p65 (Ser536), RelB, Akt, phospho-Akt (Ser473), and phospho-Akt (Thr308) antisera were purchased from Cell Signaling Technology (Danvers, MA, USA); CD45, Iba-1, GFAP, γ-H2AX, HDAC4, MAP2, GAPDH, 8-oxoguanine, and Ki67 antisera were purchased from Abcam (Cambridge, MA, USA); and cyclin A1 and c-Rel antisera were purchased from Santa Cruz Biotechnology (Dallas, Texas, USA). Secondary antisera conjugated with fluorescent Alexa dye 488 and 647 and Cy3 were purchased from Life Technologies and Jackson ImmunoResearch (West Grove, PA, USA). HRP-conjugated secondary antibodies were purchased from Cell Signaling Technology and Life Technologies.

### Annexin V/propidium iodide apoptotic assay

Apoptotic and necrotic events in cell culture were assayed by annexin V/propidium iodide (V13245, Life Technologies) following the manufacturer’s protocol. In brief, coverslips were washed with cold PBS and immediately incubated with working solution containing propidium iodide and Alexa Fluor® 488 annexin V diluted in an annexin-binding buffer for 15 min. After washing with annexin-binding buffer, coverslips were mounted with anti-fading fluorescence media from Vector Laboratories (Burlingame, CA, USA).

### Immunocytochemistry and immunofluorescence

Immunocytochemistry was performed on mouse brain cryosections or PFA fixed cells according to standard methods [25]. Briefly, sections or cells were blocked in PBS containing 0.3% Triton X-100 and 10% donkey serum for 1 h in room temperature. They were then incubated in the same solution with primary antibodies overnight at 4 °C and immersed in fluorescent secondary antibodies for 1 h at room temperature. After counterstaining with DAPI for 5 min, all sections were mounted with anti-fading fluorescence media (Vector Laboratories) under a glass coverslip. All coverslips were mounted on glass slides. Experiments were the results of triplicate cultures established on separate days.

### Cell counting and Iba1/GFAP analysis

The method for cell counting was described previously [24]. Briefly, five fields were randomly chosen at × 200 final magnification on an Olympus fluorescent microscope and every neuron was counted in the images from immunohistochemistry and immunocytochemistry. The percentage of the positive PCs and cultured neurons with markers of interest were counted and expressed as a fraction of the total MAP2-positive PCs/neurons. For cell counting in the frontal cortex, total MAP2-labeled neurons within layers II to V that co-localized with markers of interest were counted at the same magnification. Iba1 and GFAP signals were analyzed in three images/animal using ImageJ software (National Institutes of Health). The threshold value was set at ~ 40 in the measurement tool, and the percentages of Iba1/GFAP occupied areas in each image were measured. Iba1 and GFAP staining factors were normalized to the untreated *Atm*^+/+^ group and expressed in a normalized ratio.

### Quantitative real-time PCR

Microglia, THP-1 cells, and mouse cerebellar tissues were lysed in buffer containing 3% β-mercapethanol; total RNA was then extracted and reverse-transcribed as previously described [40]. Real-time PCR was performed using SYBR Premix Ex Taq (Takara Biotechnology) in the 7500 Real-Time PCR System (Applied Biosystems, Life Technologies). ROX II was applied as the reference dye. The sets of primers used are listed in Tables 1 and 2. Expression levels of *Gapdh* or *18SRNA* were used for normalization.

**Table 1:** Primer for *Mus musculus*

	Forward	Reverse
*Ym1*	cagctgggatcttcctacca	attctgcattccagcaaagg
*Trem2*	ctggaaccgtcaccatcact	aggctagaggtgacccacag
*Il1β*	gccaccttttgacagtgatgag	aaggtccacgggaaagacac
*Tnfα*	aggcactcccccaaaagatg	ccacttggtggtttgtgagtg
*iNOS*	acagggagaaagcgcaaaac	gaacattctgtgctgtcccag
*Il6*	agacaaagccagagtccttcag	tgtgactccagcttatctcttgg
*Ccl2*	gctgtagtttttgtcaccaagctc	agtgcttgaggtggttgtgg
*Il12*	ctcacccttaggacccagga	ctcacccttaggacccagga
*Gapdh*	ggagaaacctgccaagtatga	ggtcctcagtgtagcccaag

**Table 2:** Primer pairs for *Homo sapiens*

	Forward	Reverse
NFκB1 (P50/P105)	cctggatgactcttgggaaa	tcagccagctgtttcatgtc
RelA (P65)	ggcgagaggagcacagatac	ctgatagcctgctccaggtc
COX2	ctgttgcggagaaaggagtc	tcatggaagatgcattggaa
IL1β	cagccaatcttcattgctca	gcatcttcctcagcttgtcc
TNFα	tccttcagacaccctcaacc	aggccccagtttgaattctt
IL6	aaagaggcactggcagaaaa	caggggtggttattgcatct
IL8	ggtgcagttttgccaaggag	ttccttggggtccagacaga
CD45	ggcagacaccagaattggtt	gggagaaagggagtggaaag
CD11B	agaacaacatgcccagaacc	gcggtcccatatgacagtct
SOCS3	caagaagccaaccaggagag	gttcagcattcccgaagtgt
18SRNA	tgcatgtctaagtacgcacggcc	gatagggcagacgttcgaatggg

### Western blot analysis

Western blots were performed on mouse cerebellar tissues according to standard methods [40]. Tissue lysates were prepared in RIPA lysis buffer (Millipore, Billerica, MA, U.S.A) containing protease and phosphatase inhibitors (Roche, Grenzacherstrasse, Basel, Schweiz). Protein samples were separated by gel electrophoresis, transferred to nitrocellulose membrane (Bio-Rad), and blocked with 5% albumin bovine serum (Sigma-Aldrich) or 5% milk (Bio-Rad). After incubation with primary antibodies at room temperature overnight and then secondary antibodies at room temperature for 1 h, protein signals were visualized using ECL substrate reagents (Thermo Scientific, Waltham, MA, USA). The intensities of the bands were quantified by ImageJ and normalized to the GAPDH level.

### ELISA

ELISA kits against TNFα (#DY410) and IL1β (#DY401) were purchased from R&D systems. Cerebellar protein lysate was diluted in 1:10, and protein levels of TNFα and IL1β were determined according to the manufacturer’s protocol. Both forms of IL-1 β, pro-IL-1 β, and cleaved-IL-1β were detected by the ELISA kit. However, the signals were more specific to the cleaved form. Quantification was performed based on the cleaved form of IL-1 β.

### Statistics

Student’s unpaired *t test* and two-way ANOVA with Bonferroni post hoc test (Prism, GraphPad software, Version 7) were used to determine the differences in values between different groups. *p* < 0.05 was considered statistically significant. These are the symbols used for significance in the figures: */#: *p* < 0.05, **/##: *p* < 0.01, ***/###: *p* < 0.001.

### Study approval

All animals were housed at the Animal and Plant Care Facility of Hong Kong University of Science and Technology. All procedures involving animals were approved by the Department of Health, Hong Kong. In the writing of the article, every effort has been made to follow the ARRIVE guidelines (https://www.nc3rs.org.uk/arrive-guidelines).

## Results

### Microglia-mediated immune challenge contributes to the neuronal damage in vitro

**Fig. 1:**
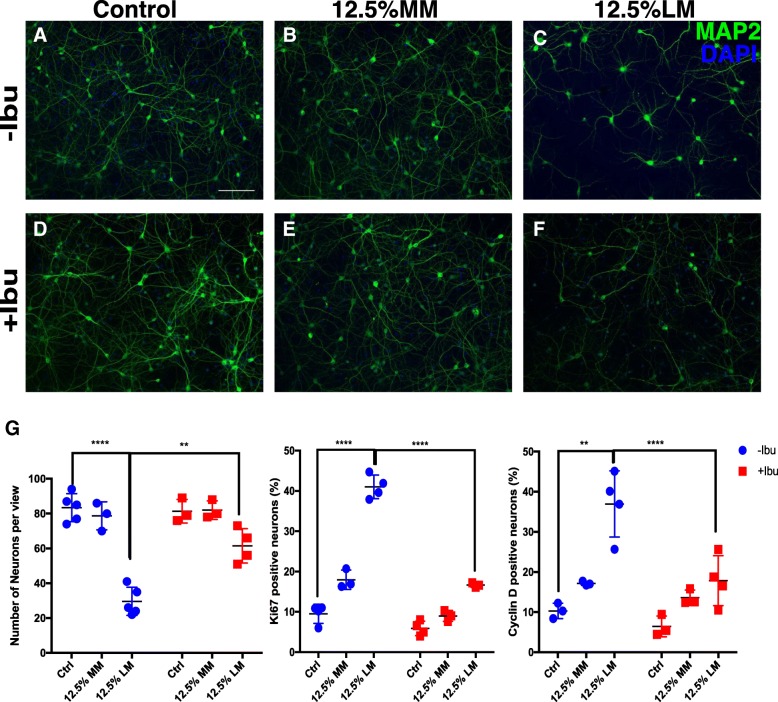
Beneficial effect of ibuprofen seen as neuroprotection after inflammatory stress. Cortical neurons with or without ibuprofen pre-treatment were treated with MM or LM (12.5%) for 24 h then fixed and processed for immunocytochemistry. Control, MM, and LM treated groups are shown in panel (**a**–**c** and **g**). Ibuprofen pre-treated groups are shown in panels **d**–**f** and **g**. Quantification confirmed the above findings and their significance was determined with a two-way ANOVA (G and Table 3). Scale bar = 50 μm. *n* = 3–4 for each group. ** *p* < 0.01, *** *p* < 0.001, **** *p* < 0.0001 between control and LPS/MM/LM-treated groups without ibuprofen treatment

Previous studies in our lab have found that an LPS (lipopolysaccharide) immune challenge significantly exaggerates neuronal damage in both the short and long-term [24, 25]. To replicate this effect in an in vitro system, we established enriched cultures of microglia from wild-type mice, applied LPS (lipopolysaccharide) directly to the cultures (10 μg/ml for 48 h), harvested the medium, and applied it to separate cultures of the neuron. This treatment was effective as the LPS-conditioned medium (LM) caused the exposed neuronal cells to undergo a marked morphological change (Fig. 1). In addition, the neurons demonstrated enhanced stress. They showed significant cell loss as measured by decreased MAP2 counts and increased cell cycle activity as measured by both enhanced Ki67 and cyclin D staining. This was not an effect of residual LPS in the LM; direct treatment of neuronal cultures with 10 μg/ml LPS (Table 3) failed to induce any of these symptoms of neuronal damage. A measurable neuronal response was also seen with conditioned medium from un-stimulated microglial cultures (MM), as high concentrations of MM (12.5%) induced a slight degree of damage to the cultured neurons [41]. Nonetheless, neurotoxicity was significantly exaggerated when microglia were pretreated with LPS; this shows that under a specific immune challenge, more harmful substances were released into the medium [42]. To more closely mimic the chemistry of the human immune response, we repeated these experiments with the human THP-1 monocyte cell line instead of primary mouse microglia [43–45]. This treatment also upregulated the expression of pro-inflammatory genes (Additional file 1: Figure S1). Further, just as with the medium from primary mouse microglial cells, both unstimulated (TM) and LPS-stimulated (LM) conditional media from THP-1 cells caused neurotoxicity and ectopic cell cycle reentry (Additional file 2: Figure S2A-N).

**Table 3:** Two-way ANOVAs analysis for cyclin D and Ki67 counts in all treatment groups

	Cyclin D *p* value	Ki67 *p* value
Control vs LPS	**< 0.05**	> 0.05
Control vs 12.5% LM	**< 0.001**	**< 0.0001**
12.5% LM vs 12.5% LM		
+ 200 μM ibuprofen	**< 0.0001**	**< 0.0001**
Control vs 6.25% LM	**< 0.001**	**< 0.001**
6.25% LM Vs 6.25% LM		
+ 200 μM ibuprofen	**< 0.001**	**< 0.001**
Control vs 6.25% MM	**< 0.05**	> 0.05
6.25% MM Vs 6.25% MM		
+ 200 μM ibuprofen	> 0.05	> 0.05
Control vs 12.5% MM	**< 0.05**	**< 0.05**
12.5% MM Vs 12.5% MM		
+ 200 μM ibuprofen	**< 0.05**	**< 0.05**

Samples that are statistically significant were shown in boldface in the table

**Fig. 2:**
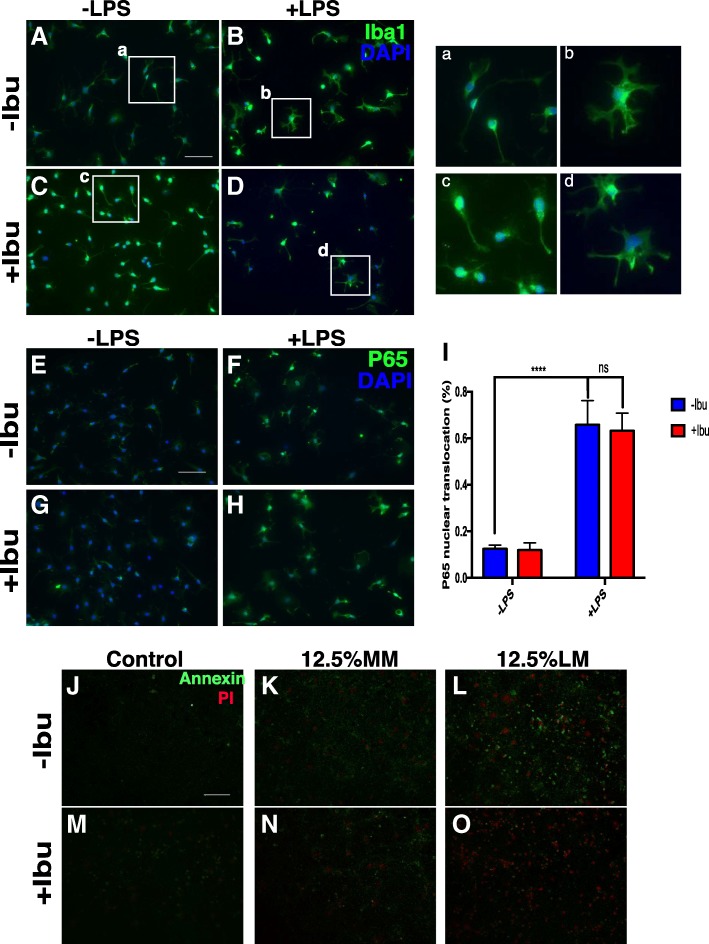
**a**, **b** LPS challenge dramatically changes the morphology of microglia and induces the activation of the NF-κB pathway (**e, f**, and **i**). **c, d, g**–**i** Ibuprofen did not block the activation of microglia after LPS stimulation. **j**–**o** Both annexin V and PI signals accumulated in neuronal culture when treated with LM. Ibuprofen pretreatment significantly blocked both apoptotic and necrotic processes. Scale bar = 50 μm. *n* = 4 for each group. ** *p* < 0.01, *** *p* < 0.001, **** *p* < 0.0001

Given the fact that direct application of LPS does not damage cells in neuronal cultures, these data demonstrate that it is the products of microglia, responding to the LPS challenge, that cause the damage. To determine the nature of the neurodegeneration caused by conditioned media, neurons were stained with annexin V/propidium iodide [46]. With these reagents, apoptotic cells fluoresce green while cells that die by non-apoptotic means show red plus green fluorescence. Live cells show little or no fluorescence (Fig. 2j–o). In cultures treated only with MM, cells were only lightly labeled with annexin V (green) suggesting that the deaths were largely apoptotic events. No obvious cell death of any kind was identified in control groups (Fig. 2j). Under 12.5% LM treatment, however, most cells showed strong green plus red signals (Fig. 2l). This suggests that LM kills neurons via a non-apoptotic, possibly necrotic process (see also Fig. 1).

### Ibuprofen attenuates inflammatory stress and cell death

To determine whether ibuprofen could be effective at reducing the complex inflammatory stress caused by activation of the innate immune system, DIV14 cortical neurons were pretreated with ibuprofen 24 h before the MM or LM challenge. Cells were then stained with cell cycle markers to assess neuronal stress. ANOVA revealed a significant effect of ibuprofen treatment on ectopic cell cycle reentry with significant conditioned medium cross ibuprofen interaction. (Fig. 1 and Additional file 2: Figure S2A–N) Ibuprofen treatment alone did not induce any significant changes in control cultures or neuronal cultures subjected to direct LPS treatment (Fig. 1 and data not shown). In the more complex environment of LM conditioned medium, however, post hoc tests showed that ibuprofen treatment suppressed LPS-induced neuronal cell cycle events (CCEs) (Fig. 1g, Additional file 2: Figure S2I–N and Table 3). Notably, 120 μM ibuprofen significantly reduced neuronal apoptosis as measured by the annexin V/PI signal (Fig. 2m–o). Thus, when applied directly to neurons, ibuprofen has a significant neuroprotective effect against LM-induced neuroinflammation.

Interestingly, ibuprofen pretreatment of the microglial themselves did not block their activation by LPS nor their ability to produce neurotoxic material in their medium (Fig. 2a–i). LPS induced a substantial morphological change from a resting ramified state to a more amoeboid-like active stage. To our surprise, however, pretreating the microglia with ibuprofen for 12 h (Fig. 2c, d, g, h) did not block the effect of LPS and had little impact on the activation of the immune pathway in microglia (Fig. 2e–i). After LPS stimulation, the percentage of cells with an activated NF-κB pathway, as measured by the translocation of p65 from the cytoplasm to the nucleus, significantly increased, but pretreatment with ibuprofen failed to block this response. We speculate that, given the long-term nature of our assay, these results are most likely due to the chronic versus acute nature of the inflammatory response that we measured [47]. Our data therefore demonstrate that ibuprofen can provide immunoprotection to neurons if applied directly and this effect is independent of any direct effect of ibuprofen on LPS-stimulated microglia.

### Ibuprofen improves LPS-induced symptoms in *Atm*^−/−^ mice

**Fig. 3:**
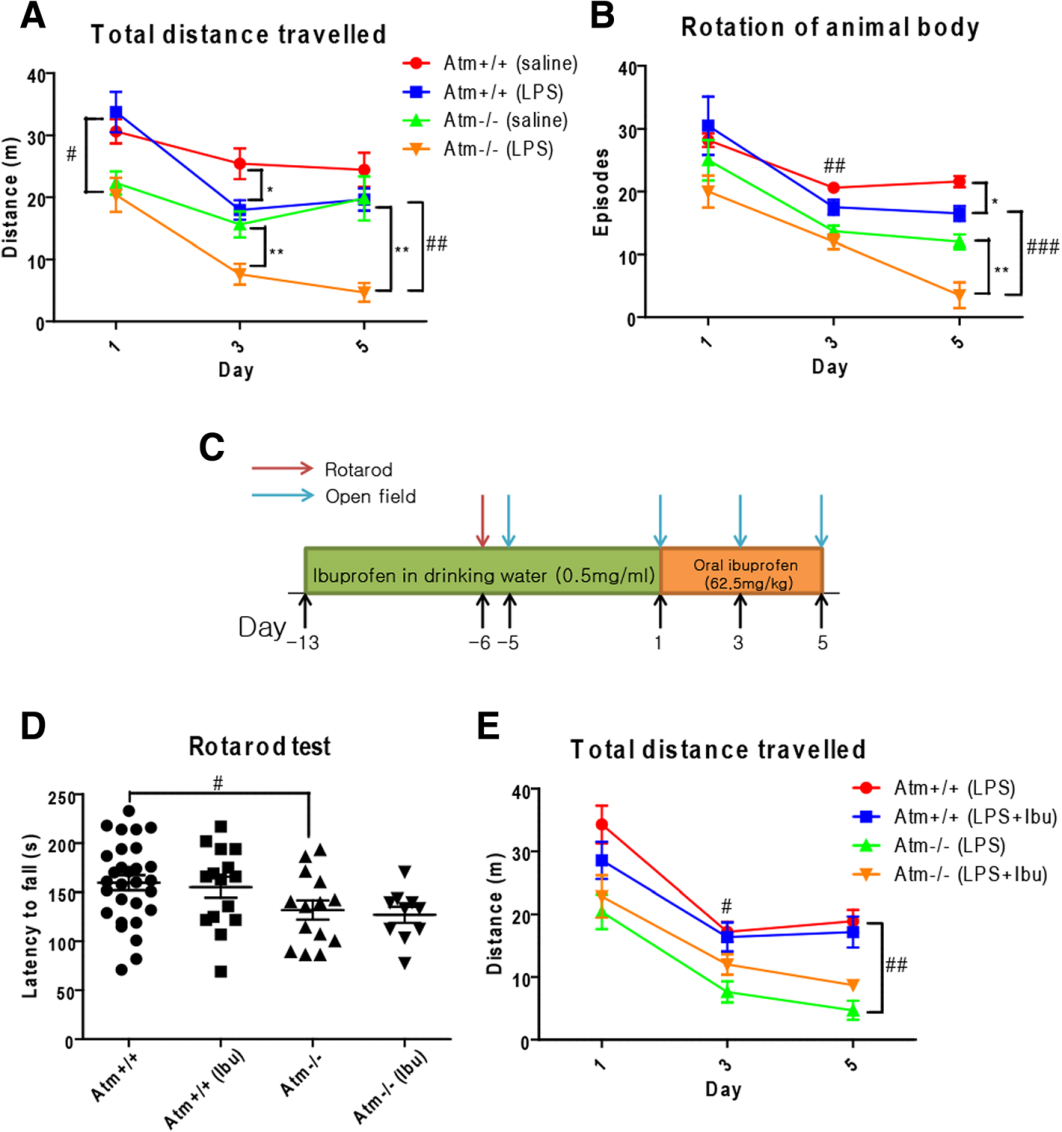
Rotarod and open field test on *Atm*^+/+^and *Atm*^−/−^ animals with 2-week ibuprofen administration followed by 4-day LPS injection. *Atm*^−/−^ animal traveled less distance (A) on day 1 before LPS administration. After LPS injection, both genotypes showed a significant loss of motor activity as measured by distance traveled (**a**) and rotation (**b**) on day 3 although some recovery was noted at day 5. *n* = 6–10 for each group. **c** Experimental timeline for ibuprofen experiment. **d** ATM deficiency significantly reduced rotarod performance of *Atm*^−/−^ animal and ibuprofen failed to improve it. **e** Similar results were observed in open field tests; ibuprofen failed to improve reduced motor activities in LPS injected *Atm*^−/−^ animals. *n* = 8–11 for each group. Two-way ANOVA was used for analyzing differences in genotype and ibuprofen treatment within an individual day. * *p* < 0.05, ** *p* < 0.01 between saline and LPS groups within the same genotype; # *p* < 0.05, ## *p* < 0.01, ### *p* < 0.001 between *Atm*^+/+^ and *Atm*^−/−^ groups with the same treatment

In vivo ATM deficiency exacerbates the neuronal damage induced by LPS-triggered bouts of neuroinflammation [24]. To test the effects of ibuprofen on this response, we administered LPS by i.p. injection to 3-month-old *Atm*^+/+^ and *Atm*^*−/−*^ mice for 4 days. Mice from all treatment groups were then subjected to behavioral testing. Two days after its initiation, ANOVA revealed significant effects of the LPS treatment (distance traveled: [*F*(1, 27) = 12.12, *p* = 0.0017], body rotation: [*F*(1, 27) = 8.91, *p* = 0.0114]) and genotype (distance traveled: [*F*(1, 27) = 20.35, *p* = 0.0001], body rotation: [*F*(1, 27) = 36.37, *p* < 0.0001]). LPS injection led to reduced motor behavior as seen by both reduced total travel distance (*Atm*^*+/+*^*: p* < 0.05; *Atm*^*−/−*^: *p* < 0.01—Fig. 3a) and reduced number of body rotations (Fig. 3b). The LPS-induced behavioral deficiency, observed at day three, remained stable at day five in the wild-type (*Atm*^+/+^) animals, but continued to decline in the *Atm*^−/−^ mutant mice (distance traveled: [*F*(1, 27) =14.48, *p* = 0.0007], post hoc test: *p* < 0.01; body rotation: [*F*(1, 27) =23.35, *p* = 0.0004], post hoc test: *p* < 0.01) (Fig. 3a–b). Thus, an LPS challenge worsens many of the AT neurological phenotypes and exacerbates the subtle motor deficiency of the AT mouse model.

We then tested whether ibuprofen might have beneficial effects in blunting an LPS-induced inflammatory challenge in vivo. The experimental procedure is diagrammed in Fig. 3c. Three-month-old *Atm*^+/+^ and *Atm*^−/−^ animals were administered ibuprofen (0.5 mg/ml in their drinking water) for 2 weeks. Animals were subjected to rotarod and open field tests after 1 week of ibuprofen treatment. One week later, we began a daily regimen of intraperitoneal injections of LPS (1 mg/kg) for 4 days. During this final phase, we tested mice in the open field every 2 days. We confirmed the compromised motor performance of the *Atm*^−/−^ animals on the rotarod (Fig. 3d—[*F*(1, 64) = 7.80, *p* = 0.0069], post hoc test: *p* < 0.05 by ANOVA) and open-field tests (Fig. 3a—[*F*(1, 27) = 14.05, *p* = 0.0009], post hoc test: *p* < 0.05) and showed that LPS injection further degraded the performance of both mutant and wild-type animals in the open field test. Unfortunately, a 2-week ibuprofen treatment of *Atm*^+/+^ mice was unable to block the LPS effect, consistent with its modest long-term effect on the microglial reaction. Although the motor behavior performance of *Atm*^−/−^ animals continued to decline, ibuprofen led to a modest lessening of the LPS-induced deterioration (Fig. 3e). There was little effect of the NSAID on the baseline behavior of *Atm*^*−/−*^ mice (Fig. 3d, e) although open field behavior improved slightly on day five.

### Ibuprofen promotes anti-inflammatory and anti-oxidative effects in ATM-deficient conditions

**Fig. 4:**
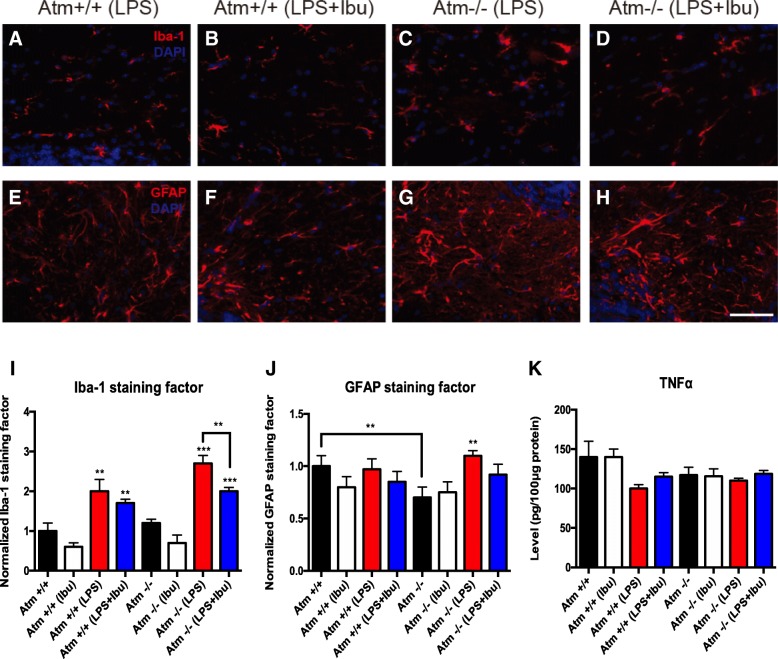
*Ibuprofen* suppressed LPS-induced inflammation in *Atm*^−/−^ cerebellum. **a**–**d** Microglia staining. **e**–**h** Astrocytes staining. LPS specifically induced higher microglial (**c**) and astrocytic (**g**) activation in *Atm*^−/−^ deep cerebellar nuclei, while ibuprofen supplementation reversed this effect (**d**, **h**). Quantification shows the same trend even though significance was achieved for microglial (**i**) but not astrocytic (**j**) cells. TNFα and IL1β levels in the whole cerebellum were measured by ELISA (**k**, **l**). Scale bar = 50 μm. *n* = 4 for each group. Student’s unpaired *t test* was used to analyze the difference between *Atm*^+/+^ and *Atm*^−/−^ groups without any treatments. Two-way ANOVA was used for analyzing differences in LPS and ibuprofen treatments within the same genotype. ** *p* < 0.01 as indicated in graphs; ## *p* < 0.01, ### *p* < 0.001 between saline and LPS groups within the same genotype

While the behavioral deficits were largely refractory to change, we found important anti-inflammatory and anti-oxidative effects of ibuprofen at the cellular level. At this resolution, the effects of chronic LPS treatment on brain microglia were apparent, as assessed by Iba1 staining and morphology (Fig. 4i). LPS administration significantly induced microglia activation in both wild-type and *Atm*^−/−^ mice. We quantified the Iba1 staining factor by measuring the area occupied by Iba1-positive glia and dividing by the total area of the image. The deep cerebellar nuclei (DCN) represent the final output of the cerebellar circuitry and the hyperexcitability of this region leads to cerebellar ataxia when there is a loss of inhibitory inputs from cortical Purkinje cells [48]. We therefore focused our analysis on this region. ANOVA revealed a significant effect of LPS treatment on both genotypes (*Atm*^+/+^: [*F*(1, 12) = 33.46, *p* < 0.0001], *Atm*^−/−^: [*F*(1, 12) = 125.93, *p* < 0.0001]) but the effect of ibuprofen was seen only in *Atm*^−/−^ animals [*F*(1, 12) = 19.61, *p* = 0.0008]. Iba1 microglial staining increased after LPS injection, and this reaction could be suppressed by ibuprofen treatment in both genotypes (Fig. 4a–d, i). This effect reached significance only in *Atm*^*−/−*^ animals pre-treated with ibuprofen. Examined before the immune stimulus, *Atm*^−/−^ cerebellar astrocytes showed evidence of reduced activation as assessed by GFAP immunostaining (Fig. 4j). LPS was able to activate astrocytes in the *Atm*^−/−^ mutant cerebellum ([*F*(1, 10) = 21.67, *p* < 0.0001], post hoc test: *p* < 0.001) but not in *Atm*^+/+^ animals, consistent with our previous work [24]. Even in *Atm*^−/−^ mutants, however, ibuprofen failed to reduce the astrocytic response to LPS (Fig. 4e–h, j).

In chronic inflammatory situations lasting days, rather than hours, TNFα exposure has a neuroprotective role while chronic IL1β stimulates neurodegeneration in *Atm*^−/−^ cerebellum [25]. To determine the effects of ibuprofen treatment on these two cytokines, ELISA was performed on whole cerebellar lysates. LPS and ATM deficiency alone each showed a trend towards reduced TNFα in the cerebellum (Fig. 4k). A decrease in ATM-deficient cells would be consistent with our previous observations and could partially explain how inflammation and ATM deficiency accelerate cerebellar damage [24]. We note that the ibuprofen pretreatment regimen failed to change even the trend in TNFα levels in both *Atm*^+/+^ and *Atm*^−/−^ cerebella (Fig. 4k).

Recent studies have demonstrated that peripheral immune cells also contribute to neurodegeneration in several CNS disorders [49–52]. This led us to look for infiltration of proliferating monocytes using CD45 immunohistochemistry. LPS significantly increased monocyte infiltration (Additional file 3: Figure S3A, C, E, G) in the cerebellum from both genotypes. The expression of *Ym1* and *Trem2*, two genes associated with the inhibition of inflammation and restoration of homeostasis [53, 54], were upregulated in both genotypes after LPS challenge (*Trem2*: *Atm*^*+/+*^*:* [*F*(1, 8) = 118.67, *p* < 0.0001], post hoc test: *p* < 0.001; *Atm*^*−/−*^: [*F*(1, 8) = 230.22, *p* < 0.0001], post hoc test: *p* < 0.001) (*Ym1*: [*Atm*^*+/+*^*: F*(1, 8) = 9.44, *p* = 0.0153], post hoc test: *p* < 0.05; *Atm*^*−/−*^: [*F*(1, 8) = 8.97, *p* = 0.0172], post hoc test: *p* < 0.05)) (Additional file 2: Figure S2I and J). Ibuprofen treatment suppressed the LPS-induced monocyte infiltration and further upregulated *Trem2* expression in the remaining CNS monocytes in *Atm*^−/−^ cerebellum ([*F*(1, 8) = 23.98, *p* = 0.0012], post hoc test: *p* < 0.001) (Additional file 2: Figure S2C, D, G, H, J), suggesting a possible mechanism for the anti-inflammatory effect of ibuprofen in neurodegenerative disease.

**Fig. 5:**
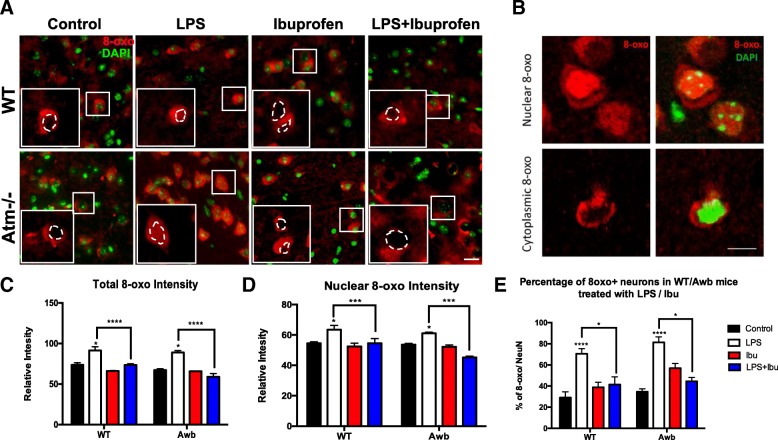
Ibuprofen blocked LPS induced oxidative stress in both wild-type and Atm^−/−^ mice. **a** DNA oxidation was identified by 8-oxo-dG immunohistochemistry. The nuclear and cytoplasmic intensity of the 8-oxo-dG signal was quantified separately **b** Confocal images of neurons demonstrating that the nuclear and cytoplasmic signal of 8-oxo-dG invidiously. The second type of 8-oxo cellular distribution pattern was shown in the lower panel with a clean cut of the nuclear region. **c** Relative total intensity measured by ImageJ. **d** Relative nuclear intensity measured by ImageJ. **e** Percentage of 8-oxo positive neurons, which includes both types of 8-oxo-dG patterns in Fig. 5b, in MAP2 and NeuN double-positive cells (data not shown). Scale bar = 10 μm. *n* = 3–4 for each group. Two-way ANOVA was used for analyzing differences in the effects of LPS injection and ibuprofen within the same genotype. * *p* < 0.05 as indicated in the graphs

Immunohistochemistry was performed to assess the anti-oxidative effect of ibuprofen. As shown in Fig. 5a, c, *Atm*^−/−^ cells contain higher levels of DNA oxidation as measured by 8-Oxo-2′-deoxyguanosine (8-oxo-dG), and LPS administration drives up both the total 8-oxo-dG signal intensity as well as the percentage of 8-oxo-dG positive neurons in both wildtype and *Atm*^−/−^ mice (Fig. 5a, c, e). Interestingly, not only did the total intensity of 8-oxo-dG increase in the LPS treated mice but also its nuclear localization. The nature of the cytoplasmic signal is not clear, but the enhanced levels of oxidized DNA in the cell nucleus is further evidence of the significant oxidative stress in the cells experience in these conditions and may help explain the appearance of neuronal cell cycle reentry [55] (Fig. 5a, b, d). Ibuprofen was able to blunt the LPS-induced DNA oxidation in both wildtype and ATM-deficient mice, suggesting its neuroprotective effects might be due to its function as an antioxidant. The in vivo effect was incomplete, however, as the rescue was only partial (Fig. 5). Our in vitro analysis confirmed the anti-oxidative effect of ibuprofen (Additional file 2: Figure S2O). LM treatment dramatically increased the intensity of 8-oxo-dG in cultured neurons while MM had little effect. Pre-incubation with ibuprofen significantly reduced the oxidative stress induced by LM in neuronal culture. Thus, ibuprofen alleviates the inflammatory stress caused by an LPS challenge and further mitigates the neuronal cell loss in both wild-type and *Atm*^−/−^ mice.

### Ibuprofen reduces cellular pathology in Atm^−/−^ cerebellum

**Fig. 6:**
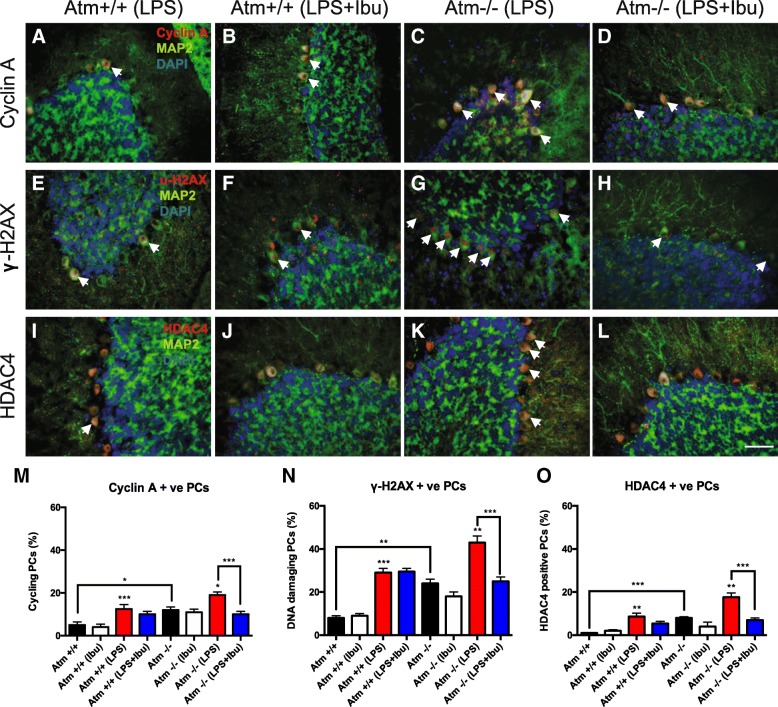
Ibuprofen protects the ATM-deficient brain by reducing Purkinje damage. Representative images of cyclin A (**a**–**d**), γ-H2AX (**e**–**h**) and nuclear HDAC4 (**i**–**l**) in WT *Atm*^−/−^ PCs under LPS and ibuprofen treatments are shown. Under normal conditions, ibuprofen had little effect on most parameters measured (**m**–**o**). After a 4-day LPS injection, however, ibuprofen pretreatment was able to suppress all three types of damage in *Atm*^−/−^ PCs but not in *Atm*^+/+^ PCs (**a**–**l**). Quantification confirmed these results (**m**–**o**). White arrows indicate PCs with respective damage markers. Scale bar = 50 μm. *n* = 4–9. Student’s unpaired *t test* was used to analyze the difference between *Atm*^+/+^ and *Atm*^−/−^ groups without any treatments. Two-way ANOVA was used for analyzing differences in the effects of LPS injection and ibuprofen within the same genotype. * *p* < 0.05, ** *p* < 0.01, *** *p* < 0.001 as indicated; # *p* < 0.05, ## *p* < 0.01, ### *p* < 0.001 between saline and LPS injected groups with the same genotype

To monitor the neuropathological correlates of these changes, 10 μm brain sections were immunoassayed with markers of cell cycle, DNA damage, and epigenetic and oxidative stress. Two-way ANOVA analysis revealed the main effects of LPS treatment on cyclin A (*Atm*^+/+^: [*F*(1, 23) = 28.49, *p* < 0.0001]), γ-H2AX (*Atm*^+/+^: [*F*(1, 23) = 60.97, *p* < 0.0001], *Atm*^−/−^: [*F*(1, 17) = 8.86, *p* = 0.0089]) and HDAC4 (*Atm*^+/+^: [*F*(1, 23) = 15.68, *p* = 0.0006], *Atm*^−/−^: [*F*(1, 17) = 8.49, *p* = 0.0097]), and of ibuprofen treatment on cyclin A (*Atm*^−/−^: [*F*(1, 17) = 6.04, *p* = 0.0251]), γ-H2AX (*Atm*^−/−^: [*F*(1, 17) = 6.69, *p* = 0.0198]), and HDAC4 (*Atm*^−/−^: [*F*(1, 17) = 11.68, *p* = 0.0033]). Consistent with our previous data [24], at baseline, *Atm*^*−/−*^ PCs had significantly higher neuronal cell cycle events (*p* < 0.05) (Fig. 6m), DNA damage (*p* < 0.01) (Fig. 6n), and nuclear localization of HDAC4 (*p* < 0.001) (Fig. 6o) than *Atm*^*+/+*^ PCs. Unfortunately, ibuprofen had no significant therapeutic effect on these three established phenotypes. As reported, LPS-induced immune challenge increased the three markers in both *Atm*^+/+^ and mutant PCs [24] (Fig. 6m–o). Unlike the unstimulated mice, however, if given before the LPS treatment, ibuprofen significantly blunted the increase in three markers. Post hoc analysis showed that ibuprofen significantly reduced cyclin A (*p* < 0.001), γ-H2AX (*p* < 0.01), and nuclear HDAC4 (*p* < 0.001) in *Atm*^*−/−*^ Purkinje cells after LPS administration (Fig. 6m–o). These results indicate that ibuprofen counteracts the negative synergy between the *Atm* genotype and the neuroinflammatory environment, reducing the vulnerability of *Atm*^*−/−*^ Purkinje cells to LPS-induced damage.

### Ibuprofen suppresses LPS- and ATM deficiency-induced p38 phosphorylation

**Fig. 7:**
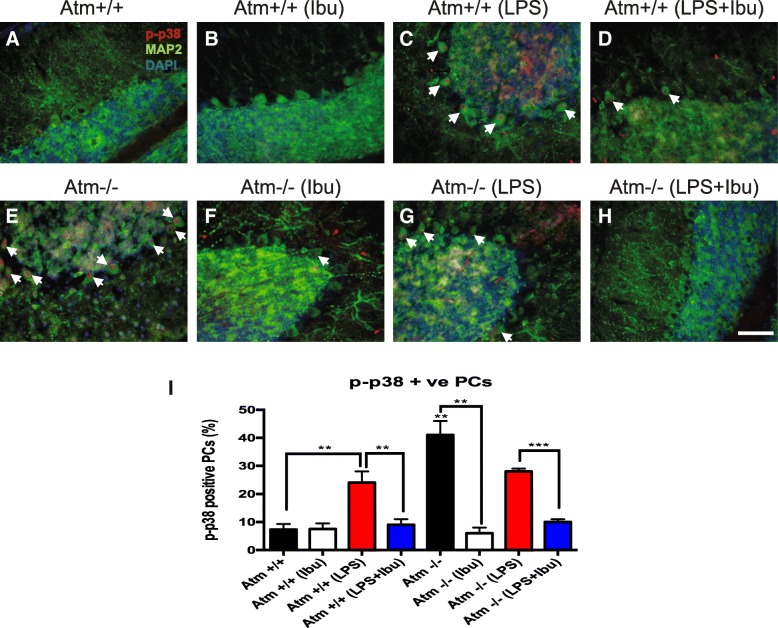
Ibuprofen specifically suppresses p38 phosphorylation in PCs. Cerebellar sections were stained with phospho-p38 antiserum and visualized by fluorescent microscopy. LPS or ATM deficiency alone increased phospho-p38 levels in PCs (**c**, **e**) yet LPS unexpectedly reduced phospho-p38 levels in *Atm*^*−/−*^ cerebellum (**g**). Ibuprofen treatment significantly reversed LPS triggered p38 phosphorylation in both *Atm*^+/+^ and *Atm*^−/−^ PCs (**d**, **h**). Quantification confirmed the results (I). White arrows indicate neurons with nuclear phospho-p38. Scale bar = 50 μm. *n* = 3 for each group. Student’s unpaired *t test* was used to analyze the difference between *Atm*^+/+^ and *Atm*^−/−^ groups without any treatments. Two-way ANOVA was used for analyzing differences in the effects of LPS injection and ibuprofen within the same genotype. ** *p* < 0.01, *** *p* < 0.001 as indicated in the graphs; # *p* < 0.05 between *Atm*^+/+^ and *Atm*^−/−^ PCs

LPS-induced neuronal damage is correlated with the activation of the MAPK, Akt, and NFκB pathways [25]. This led us to ask whether ibuprofen was able to suppress these changes. Whole cerebellar lysates were prepared, followed by Western blots with antisera to proteins involved in above three pathways. LPS, with or without ibuprofen, stimulated no obvious activation (phosphorylation) of ERK or Akt pathways in whole cerebella extracts of either genotype (Additional file 4: Figure S4). As PCs represent less than 1% of the total number of cells in the cerebellum, whole cell lysates might obscure differences that were taking place only in this one cell type. We therefore turned to immunohistochemistry to investigate the levels of phospho-p38 specifically in PCs. In this way, we found that both ATM deficiency and LPS injection in wild-type mice induced p38 activation in PCs (Fig. 7c, e and Additional file 5: Figure S5). In *Atm*^*−/−*^ mice, LPS injection did not show any combinatorial effect. Unexpectedly, p38 induction in *Atm*^−/−^ animals trended lower after LPS injection (Fig. 7g and Additional file 5: Figure S5). Two-way ANOVA revealed significant differences in LPS (*Atm*^*+/+*^*:* [*F*(1, 8) = 66.27, *p* < 0.0001], *Atm*^*−/−*^: [*F*(1, 8) = 13.20, *p* = 0.0068]) and ibuprofen treatments (*Atm*^*+/+*^*:* [*F*(1, 8) = 44.00, *p* = 0.0002], *Atm*^*−/−*^: [*F*(1, 8) = 296.98, *p* < 0.0001]) in both genotypes. Ibuprofen was able to block the LPS-induced increase in p38 phosphorylation and surprisingly was equally effective at reducing the ATM-dependent activation of p38 in PCs (Fig. 7 b, d, f, h and Additional file 5: Figure S5). Quantification of the immunohistochemistry confirmed these results (*Atm*^*+/+*^*: p* < 0.01; *Atm*^*−/−*^: *p* < 0.001) (Fig. 7i). These data thus suggest that ibuprofen mainly delays the A-T progression at least in part through the suppression of p38 activation in the cerebellum.

### Early ibuprofen treatment delays the initiation of A-T symptoms

**Fig. 8:**
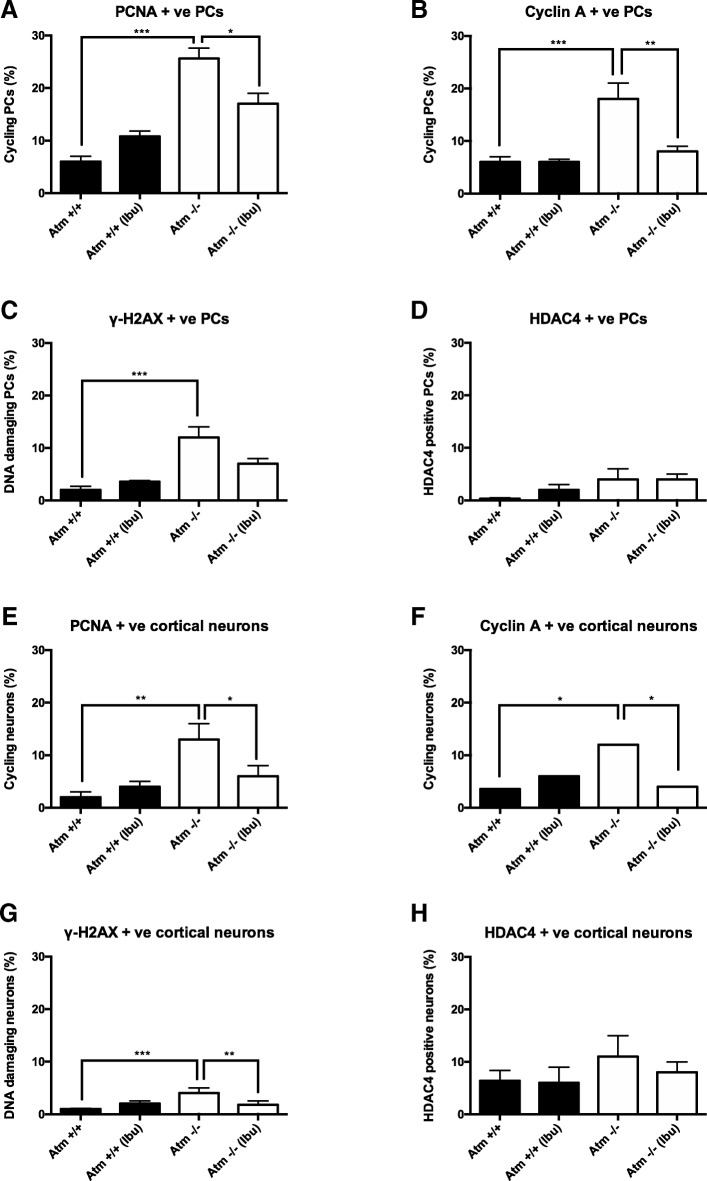
Ibuprofen reduces early cellular damage in *Atm*^*−/−*^ animals. Ibuprofen prevented cell cycle reentry (**a**–**b**) and DNA damage (**c**) in *Atm*^*−/−*^ PCs; the pattern of nuclear translocation of HDAC4 remained unchanged (**d**). Similar results were observed in *Atm*^*−/−*^ cortical neurons as measured by neuronal cell cycle activity (**e**–**f**), DNA damage (**g**) and nuclear HDAC4 expression (**h**). Ibuprofen alone did not induce any changes in *Atm*^*+/+*^ cerebellum (**a**–**d**) or cortex (**e**–**h**). *n* = 4–5 for each group. Two-way ANOVA was used for analyzing differences in genotype and ibuprofen treatment. * *p* < 0.05, ** *p* < 0.01, *** *p* < 0.001

All of the above studies were performed in adult *Atm*^*−/−*^ mice with established pathology and defined motor symptoms. We note that even at this relatively late disease stage, ibuprofen is still beneficial. We next asked whether ibuprofen might perhaps have more significant protective effects if it were applied before the first appearance of symptoms*.* To test this, wild-type and *Atm*^*−/−*^ mice were administrated ibuprofen orally for 2 weeks starting at age P10, an age before the earliest signs of PC distress are apparent [56]. After 2 weeks of treatment, the animals were killed by perfusion and their brains embedded in OCT for sectioning on a cryostat. Immunostaining was performed to access the level of neuronal damage. We found significant effects of genotype (PCNA*:* [*F*(1, 14) = 54.19, *p* < 0.0001]; cyclin A: [*F*(1, 14) = 21.23, *p* = 0.0004]) and ibuprofen treatment (cyclin A: [*F*(1, 14) = 10.82, *p* = 0.0054]) in the damage markers by two-way ANOVA. Post hoc test revealed that ibuprofen blocked the developmental appearance of ectopic cell cycling in the *Atm*^*−/−*^ Purkinje cells (PCNA: *p* < 0.01, cyclin A: *p* < 0.001— Fig. 8a, b). By contrast, DNA damage was induced in *Atm*^*−/−*^ Purkinje cells ([*F*(1, 14) = 26.64, *p* = 0.0001], post hoc test: *p* < 0.01) but not significantly reduced under ibuprofen treatment (Fig. 8c). No change was found in HDAC4 localization (Fig. 8d). The same phenomenon was observed in *Atm*^*−/−*^ neocortical neurons where we found a significant effect of ibuprofen in suppressing these damage markers under post hoc analysis (PCNA: *p* < 0.05; cyclin A: *p* < 0.05; γ-H2AX: *p* < 0.01—Fig. 8e–g). In wild-type brains, ibuprofen had no effect, perhaps because there was little to no signal to begin with, (Fig. 8a–h). Of note, we found that HDAC4 translocation to the nucleus was not obvious in 1-month-old *Atm*^*−/−*^ animals. This suggests that, although it will develop as an important phenotype by 3 months of age (Fig. 6 plus reference [57]), it is less prominent in the developing *Atm*^*−/−*^ brain.

## Discussion

The immune and nervous systems interact extensively—with each affecting the behavior of the other for both good and bad. ATM deficiency, in humans and in mice, illustrates this point well. By morphological criteria, brain microglial cells are activated in humans, *Atm*^−/^^−^ mice, and a recently developed *Atm*^−/−^ rat model and display a more severe pro-inflammatory syndrome [58, 59]. The innate immune system in ATM deficiency also appears to be hypersensitive to exogenous challenges [60, 61]. The exaggerated loss of motor function after LPS treatment of *Atm*^−/−^ mice and the correlated changes seen in our immunohistochemistry findings suggest that this hypersensitivity leads to neuronal cell stress as seen in the accumulation of neuronal cell cycle events, evidence of DNA damage, and the nuclear localization of HDAC4 in Purkinje neurons.

Recent developments make it likely that the involvement of ATM in this chronic activation of the immune system is due in part to the presence of DNA damage. Accumulated cytoplasmic DNA, presumably enhanced after DNA damage, can trigger an innate immune response [58, 59, 62, 63]. .In our model, increased oxidative stress, produced during the immune challenge, induces DNA damage in the nucleus. In the absence of ATM function, DNA repair is compromised which leads to DNA fragments accumulating in the cytoplasm. This would then serve as a “danger” signal that would initiate an immune response via the viral DNA sensor STING or other factors [59]. Thus, an immune challenge exacerbates the oxidative stress in a cell leading to more DNA damage and an increased cellular reliance on ATM function.

### Anti-oxidative effect as the main protective mechanism

The presence of oxidative stress in conditions of ATM deficiency is clear and our data suggest that it is in fact the ability of ibuprofen to function as an anti-oxidant that serves as the major source of its ability to blunt the effects of an immune challenge both in vivo and in vitro. Previous findings and our own results have shown that LPS induces cytokine expression [14, 15] and triggers the release of reactive oxygen products [16, 17] in primary cultured microglia, human THP-1 cells, and macrophages [34]. As the conditioned medium from LPS-stimulated microglia (LM) contains a “cocktail” of cytokines and ROS, it is likely that these works together to cause neuronal damage. As expected, LPS increased the neurotoxic effect of the medium from unstimulated microglia (MM) and ibuprofen was able to rescue this damage. Similar to our findings (Fig. 2 and Additional file 1: Figure S1), several groups have shown that ibuprofen fails to significantly inhibit inflammation in the cultured innate immune cells and in the monocyte-neuronal co-culture system with acute inflammation [64, 65]. Indeed, in one case study of a patient with acute endotoxemia, ibuprofen was actually found to cause significant increases of circulating pro-inflammatory TNF-α and IL-6 [66]. .Importantly, in a series of related observations, long-term ibuprofen treatment and the resulting inhibition of COX activity enhanced rather than suppressed the inflammatory phenotype of macrophages [47]. As a result, the anti-inflammatory features of ibuprofen are affected by the dose, duration of incubation, and the cell types used for the experiment, an important feature to keep in mind when clinical studies are designed.

Evidence for the role of oxidative damage in the action of ibuprofen can also be found in the response of neuronal cultures challenged by exposure to medium from LPS-stimulated cells (LM). Ibuprofen reduced the number of neurons showing signs of oxidative stress and cellular damage if it was administered directly to the neurons before they were exposed to LM (Figs. 1 and 2 and Additional file 2: Figure S2). This neuroprotective effect may be due in part to the anti-oxidative properties of ibuprofen (Fig. 5 and Additional file 2: Figure S2) as described previously [34]. Oxidative stress is believed to induce neuronal apoptosis and/or necrosis-like cell death which does not involve caspase activation [67]. The data in Fig. 2 shows that LM enhances the PI signal of our cell death marker indicating the neuronal deaths are most likely necrosis-like events. Also, ibuprofen pretreatment is able to reduce PI signal in LM treated neurons, suggesting a reduction in intracellular superoxide anion production from neurons and the anti-oxidative feature of ibuprofen [67]. The previous study showed that, even as a DNA damage repair protein, ATM can also be activated by intracellular oxidative stress. Normal function and activity of ATM is significantly crucial for keeping the redox balance in the cytoplasm [68, 69]. During the LPS challenge, immune cells like microglia and THP-1 cells produce substances that serve as reactive oxidants and greatly contribute to the neuronal cell damage. Ibuprofen pretreatment relieves the oxidative stress in the microenvironment and contributes to the homeostasis in the central nerves system. These data are consistent with work from Wilkinson et al. that ibuprofen can directly protect neurons from oxidative stress [34].

### Ibuprofen can prevent but not reverse the progression of A-T symptoms

The question that naturally arises from these findings is whether reducing this innate immune system hypersensitivity might have therapeutically beneficial effects. We previously showed in a series of studies in mouse models of Alzheimer’s disease (AD) that increasing inflammation with a broad-spectrum LPS challenge advances the timing of AD symptoms while oral NSAIDs (ibuprofen or naproxen) retard it [33]. We were encouraged by these earlier findings to test whether a regimen of NSAID administration might also prove beneficial in a mouse model of A-T. As ibuprofen can directly block LPS-induced dendritic loss and reduce spine density [51], we tested whether it might also suppress inflammation-induced A-T symptoms either in vitro or in vivo.

Based on our earlier work in mouse models of Alzheimer’s disease (AD) [33], we hypothesized that ibuprofen might prevent the emergence of A-T symptoms. This hypothesis has largely proved correct. Indeed, the similarities in the response to ibuprofen of the A-T and AD models are striking, despite the fact that they are two very different neurodegenerative diseases. In parallel with the findings in the AD mice, neither the cellular nor the behavioral symptoms of adult A-T animals are reversed with NSAID therapy. PC stress fails to improve following ibuprofen treatment, and there is no obvious improvement in behavioral test performance—either coordination as measured on the rotarod or open field behavior.

Yet, ibuprofen can blunt the effects of additional immune/oxidative challenges. Similar to AD animals with ibuprofen supplementation [33, 49, 52, 70, 71], mature A-T animals treated with ibuprofen are less vulnerable to LPS-induced motor dysfunction; more modest benefits were observed for LPS-treated *Atm*^+/+^ (Fig. 4c–e). These results indicate that ibuprofen can suppress new or accelerated symptom appearance during an inflammatory challenge, but once symptoms are established, it cannot reverse the previous damage. This is exactly the situation found when the impact of ibuprofen is tested on the chronic inflammation found in AD model mice.

It is interesting to note that while ibuprofen does not reverse the behavioral symptoms of mature *Atm*^−/−^ cerebellum, it nonetheless reduces the microglial morphological response (Fig. 4), tissue oxidation (Fig. 5), and several types of PC damage (Fig. 6). These results are consistent with other groups who found that neuronal abnormalities could be reversed by ibuprofen through anti-inflammatory and anti-oxidative effects [26–28, 34] and is in line with our tissue culture findings showing that ibuprofen applied directly to neurons blocks the effects of LM; but if applied to the microglia themselves, it has little effect. The anti-inflammatory component of the effect of ibuprofen on cerebellar Purkinje cells should be viewed in the context, cerebellar microglia have different immune responses from those in cerebral cortex and hippocampus [72], and in this difference may lie the regional variability that accompanies the clinical picture in AD as well as A-T. Thus, A-T has its most dramatic effects in the cerebellum, and neuronal damage in AD is most obvious in entorhinal cortex and hippocampus. Further support for this concept comes from bioinformatics reports showing that cerebellar microglial cells have different clusters of expressed genes compared to cortical microglia. The classical (LPS) and alternative (interleukin 4) activated pathways in cerebellar microglia are also different from those observed in other brain regions [73]. An additional contributor to this regional variation might be the fact that the cerebellum has the smallest numbers of microglia and astrocytes in vivo [74].

### Early ibuprofen treatment reverses the development of A-T symptoms

More dramatic than the protection offered against new LPS-induced symptoms in adult animals is our finding that ibuprofen prevents the development of symptoms during the natural history of the *Atm*^*tm1Awb*^ mouse A-T model. Normally, cellular symptoms such as elevated cell cycle events appear between postnatal day 10 and 20 in the mouse [56]. But beginning at P10, 2 weeks of oral ibuprofen administration totally blocked the appearance of disease symptoms in both cerebellum and cortex. These data have potentially important clinical relevance as they suggest that ibuprofen might block or retard the development A-T pathology in pre-symptomatic children. The data lead to the hypothesis that under normal conditions, established disease symptoms cannot be improved by NSAID treatment, but before they appear or worsen, an individual suffering with ATM deficiency might benefit from this simple therapeutic approach. At the very least, as we have suggested previously [24], aggressive anti-inflammatory treatment during the course of A-T could have significant benefit. More speculative for the moment, but potentially more exciting, our experiments with the pre-symptomatic ATM-deficient mice suggest that early intervention in children might postpone the onset of disease symptoms for an unknown period of time. Taken together with our current findings, the data argue that ibuprofen should be considered as a therapeutic agent in preventing the initiation of A-T.

## Conclusions

The current study shows that the severity of the symptoms of A-T is closely correlated with the level of neuroinflammation and oxidative stress in the CNS. We find that the common NSAID, ibuprofen, is able to partially prevent the exacerbated behavioral deficits induced by an acute immune system challenge and can suppress several cellular deficits observed in LPS-stressed *Atm*^−/−^ mice. The data suggest that the actions of ibuprofen include both anti-inflammatory and anti-oxidative effects. Based on our findings, we propose that early ibuprofen treatment may help to prevent or delay the progression of A-T in human patients.

## Additional files

Additional file 1: **Figure S1.** Gene expression profile of microglia and THP-1 cells under LPS challenge and ibuprofen pretreatment. THP-1 cells were treated with ibuprofen (0, 3, 10, 40, 80, 200 μM) for 6 h and then challenged with LPS for 48 h. Gene expression of *TNFα* (D), *ILIβ* (E), *IL6* (F), *COX2* (G) and *IL8* (H), *SOCS3* (I), *CD45* (J), *CD11b* (K), *P50* (L) and *P65* (M) was assessed by PCR. (A). Microglia were treated with LPS for 48 h and then assessed by qPCR. (B). qPCR analysis of microglia treated with LPS and ibuprofen. *N* = 3–4 for each group. Student’s unpaired *t test* was used to analyze the difference between vehicle and LPS treated groups. Two-way ANOVA was used for analyzing differences in LPS and ibuprofen treatments. *, *p* < 0.05, **, *p* < 0.01, ***, *p* < 0.001 between LPS group and control group without ibuprofen pretreatment. (PDF 208 kb)

Additional file 2: **Figure S2.** (A-N). Ibuprofen pretreatment of THP-1 cells partially rescued LPS-induced neuronal damage. (A-H). Conditioned medium from ibuprofen pretreated THP-1 cells was harvested and applied to DIV14 cultured neurons. Ibuprofen has positive effect on neuronal survival and partially attenuated the appearance of CCEs as measured either by EdU or Ki67. *n* = 3 for each group. Two-way ANOVA was used for analyzing ibuprofen effect within each TM/LM treatment. (O). Oxidative stress in the cultured neuron was measured by 8-oxoguanine. LM from primary cultured microglia was then applied to neuronal culture. It significant increase the level of 8-oxoguanine while ibuprofen alleviate the oxidative stress in the culture system. Scale bar = 50 μm. *n* = 3 for each group (PDF 3439 kb)

Additional file 3: **Figure S3.** Ibuprofen stimulated formation of tissue-repairing monocytes in cerebellum. LPS stimulated monocyte infiltration while ibuprofen suppressed this invasion (C, D, G, H). Although ibuprofen failed to further stimulate *Ym1* expression (I), it specifically induced *Trem2* expression in monocytes infiltrating the *Atm*^*−/−*^ cerebellum (J). Scale bar = 50 μm. *n* = 3 for each group. Two-way ANOVA was used for analyzing differences in LPS and ibuprofen treatments within the same genotype. ***, *p* < 0.001 between groups with and without ibuprofen treatment in LPS injected *Atm*^*−/−*^ cerebellum; #, *p* < 0.05, ##, *p* < 0.01, ###, *p* < 0.001 groups with and without LPS treatment in the same genotype. (TIF 2427 kb)

Additional file 4: **Figure S4.** Akt, MAPK and NFκB pathways were investigated in cerebellar lysates by immunoblotting (A, B). Ibuprofen failed to affect ERK, JNK, Akt or p65 phosphorylation during an LPS challenge (C, E, F, G and data not shown). It reduced p38 phosphorylation only in *Atm*^+/+^ cerebellum (D). *n* = 4 for each group. Two-way ANOVA was used for statistical analysis. *, *p* < 0.05 compared to *Atm*^+/+^ saline group. (PDF 771 kb)

Additional file 5: **Figure S5.** Ibuprofen specifically suppresses p38 phosphorylation in PCs. Cerebellar sections were stained with phospho-p38 antiserum and visualized by fluorescent microscopy. LPS or ATM deficiency alone increased phospho-p38 levels in PCs (C, E) yet LPS unexpectedly reduced phospho-p38 levels in *Atm*^*−/−*^ cerebellum (G). Ibuprofen treatment significantly reversed LPS triggered p38 phosphorylation in both *Atm*^+/+^ and *Atm*^−/−^ PCs (D, H). (PDF 3474 kb)

## Abbreviations

AD: Alzheimer’s disease
A-T: Ataxia telangiectasia
ATM: Ataxia telangiectasia-mutated
CCE: Cell cycle event
CNS: Central nervous system
DCN: Deep cerebellar nuclei
LM: LPS-stimulated microglia conditioned medium
LM: LPS-stimulated THP-1 cell conditioned medium
LPS: Lipopolysaccharide
MM: Unstimulated microglia conditioned medium
NSAID: Non-steroidal anti-inflammatory drug
PC: Purkinje cell
ROS: Reactive oxygen species
TM: Unstimulated THP-1 cell conditioned medium
