# Supplementary figures and images for: Ibuprofen prevents progression of ataxia telangiectasia symptoms in ATM-deficient mice
Source: J Neuroinflammation. 2018 Nov 6;15:308. doi: 10.1186/s12974-018-1338-7 (PMC6220455; doi:10.1186/s12974-018-1338-7)

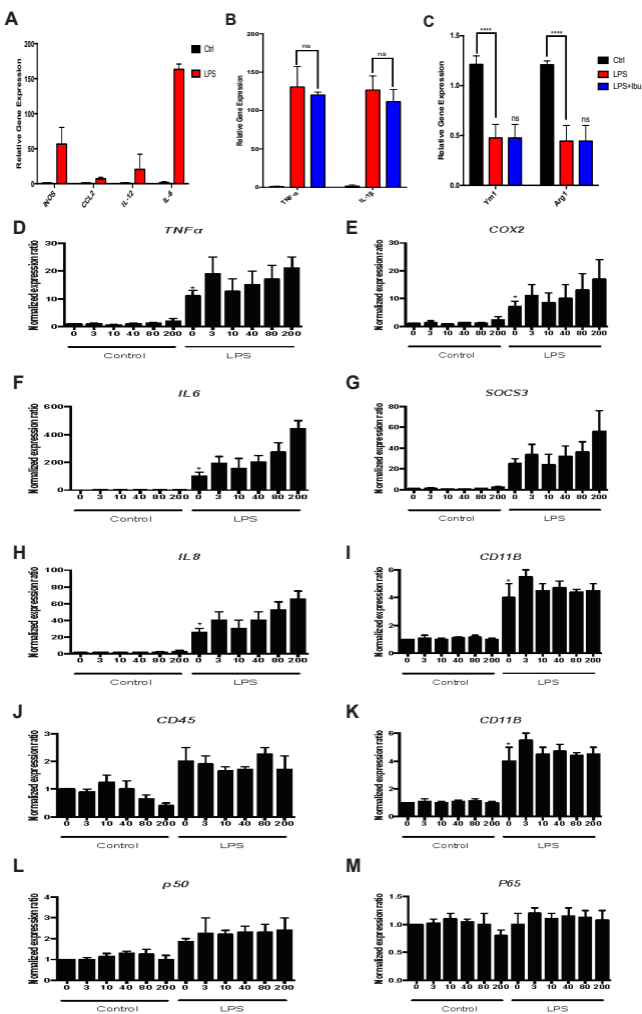

Supplement: Supplementary file 1 — Figure S1. Gene expression profile of microglia and THP-1 cells under LPS challenge and ibuprofen pretreatment. THP-1 cells were treated with ibuprofen (0, 3, 10, 40, 80, 200 μM) for 6 h and then challenged with LPS for 48 h. Gene expression of TNFα (D), ILIβ (E), IL6 (F), COX2 (G) and IL8 (H), SOCS3 (I), CD45 (J), CD11b (K), P50 (L) and P65 (M) was assessed by PCR. (A). Microglia were treated with LPS for 48 h and then assessed by qPCR. (B). qPCR analysis of microglia treated with LPS and ibuprofen. N = 3–4 for each group. Student’s unpaired t test was used to analyze the difference between vehicle and LPS treated groups. Two-way ANOVA was used for analyzing differences in LPS and ibuprofen treatments. *, p < 0.05, **, p < 0.01, ***, p < 0.001 between LPS group and control group without ibuprofen pretreatment. (PDF 208 kb) [file 12974_2018_1338_MOESM1_ESM.pdf]

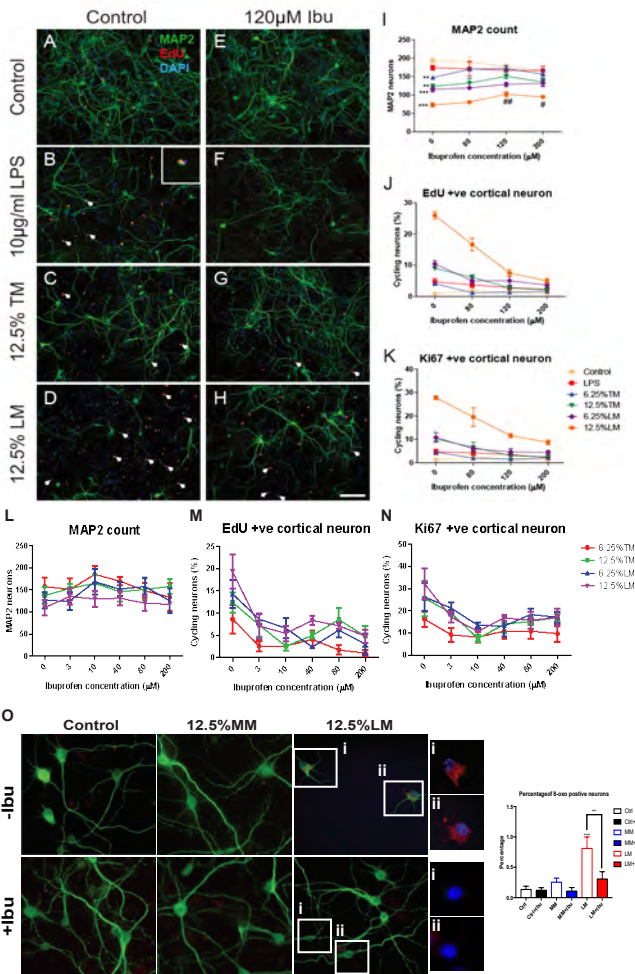

Supplement: Supplementary file 2 — Figure S2. (A-N). Ibuprofen pretreatment of THP-1 cells partially rescued LPS-induced neuronal damage. (A-H). Conditioned medium from ibuprofen pretreated THP-1 cells was harvested and applied to DIV14 cultured neurons. Ibuprofen has positive effect on neuronal survival and partially attenuated the appearance of CCEs as measured either by EdU or Ki67. n = 3 for each group. Two-way ANOVA was used for analyzing ibuprofen effect within each TM/LM treatment. (O). Oxidative stress in the cultured neuron was measured by 8-oxoguanine. LM from primary cultured microglia was then applied to neuronal culture. It significant increase the level of 8-oxoguanine while ibuprofen alleviate the oxidative stress in the culture system. Scale bar = 50 μm. n = 3 for each group (PDF 3439 kb) [file 12974_2018_1338_MOESM2_ESM.pdf]

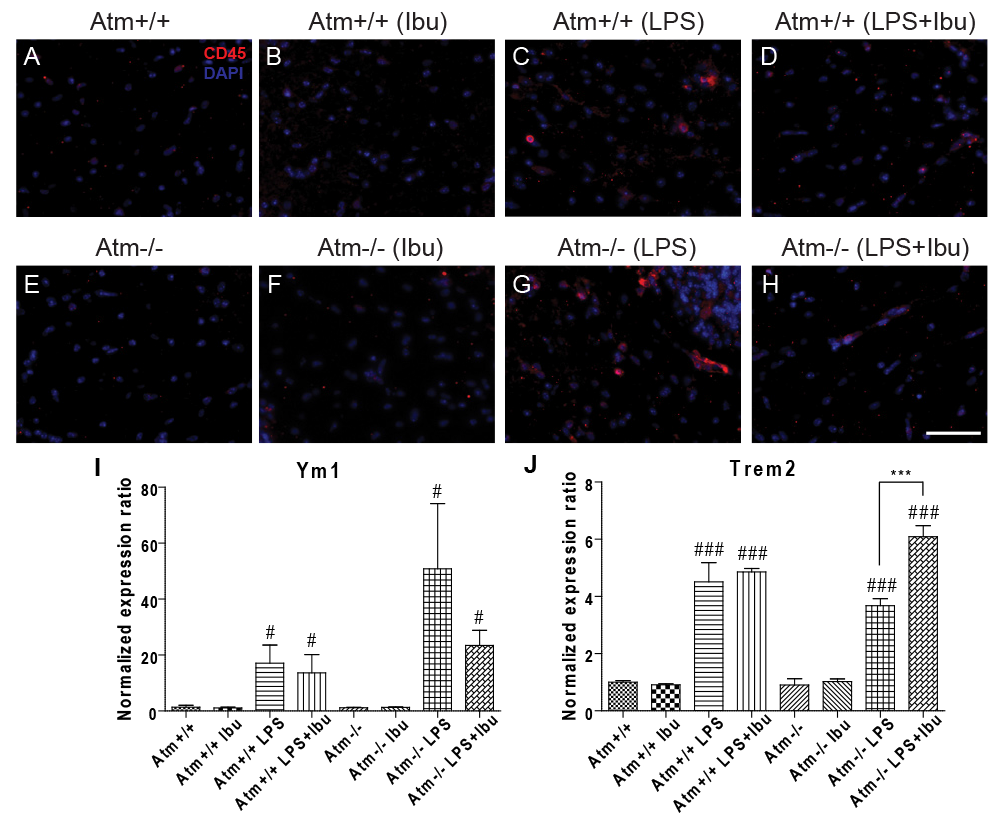

Supplement: Supplementary file 3 — Figure S3. Ibuprofen stimulated formation of tissue-repairing monocytes in cerebellum. LPS stimulated monocyte infiltration while ibuprofen suppressed this invasion (C, D, G, H). Although ibuprofen failed to further stimulate Ym1 expression (I), it specifically induced Trem2 expression in monocytes infiltrating the Atm−/− cerebellum (J). Scale bar = 50 μm. n = 3 for each group. Two-way ANOVA was used for analyzing differences in LPS and ibuprofen treatments within the same genotype. ***, p < 0.001 between groups with and without ibuprofen treatment in LPS injected Atm−/− cerebellum; #, p < 0.05, ##, p < 0.01, ###, p < 0.001 groups with and without LPS treatment in the same genotype. (TIF 2427 kb) [file 12974_2018_1338_MOESM3_ESM.tif]

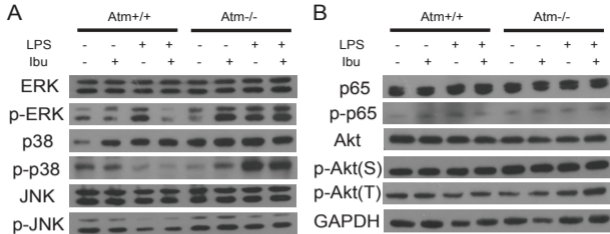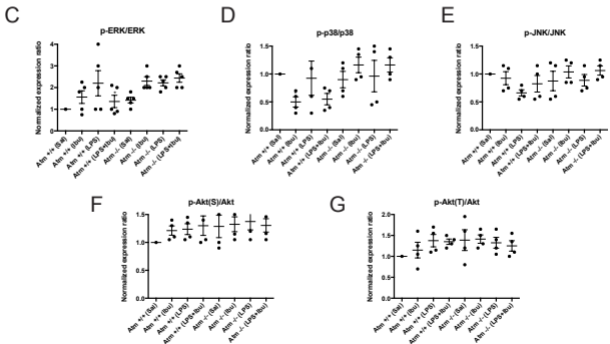

Supplement: Supplementary file 4 — Figure S4. Akt, MAPK and NFκB pathways were investigated in cerebellar lysates by immunoblotting (A, B). Ibuprofen failed to affect ERK, JNK, Akt or p65 phosphorylation during an LPS challenge (C, E, F, G and data not shown). It reduced p38 phosphorylation only in Atm+/+ cerebellum (D). n = 4 for each group. Two-way ANOVA was used for statistical analysis. *, p < 0.05 compared to Atm+/+ saline group. (PDF 771 kb) [file 12974_2018_1338_MOESM4_ESM.pdf]

**Atm+/+**

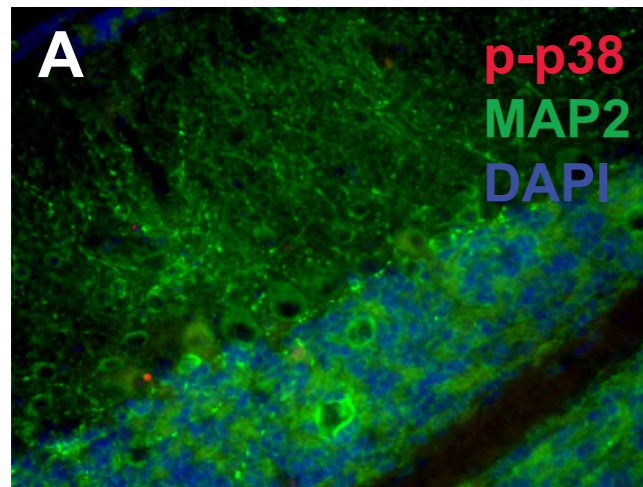

**Atm+/+ (Ibu)**

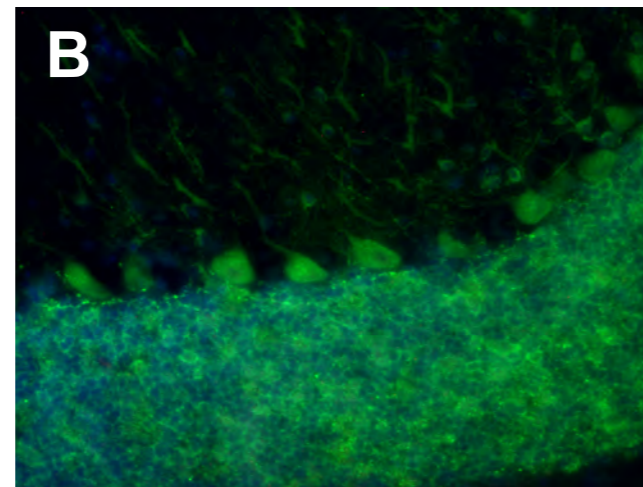

**Atm+/+ (LPS)**

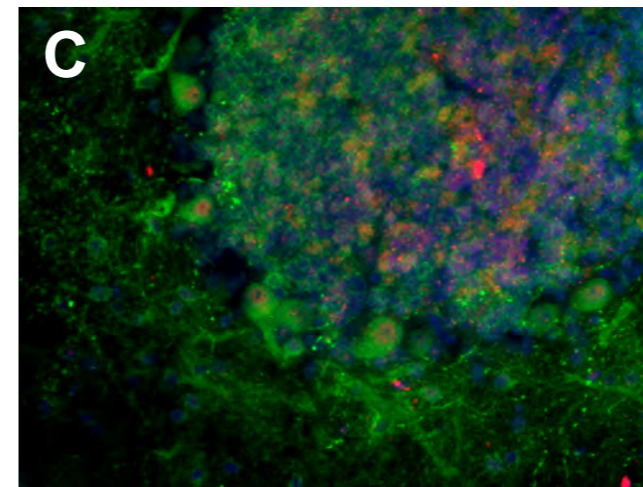

**Atm+/+ (LPS+Ibu)**

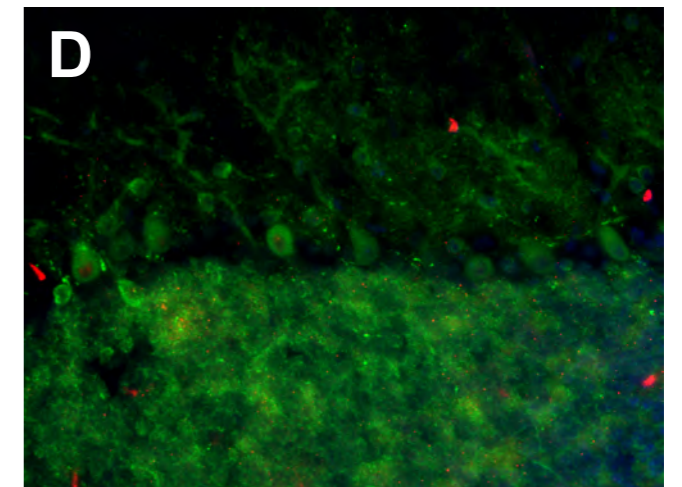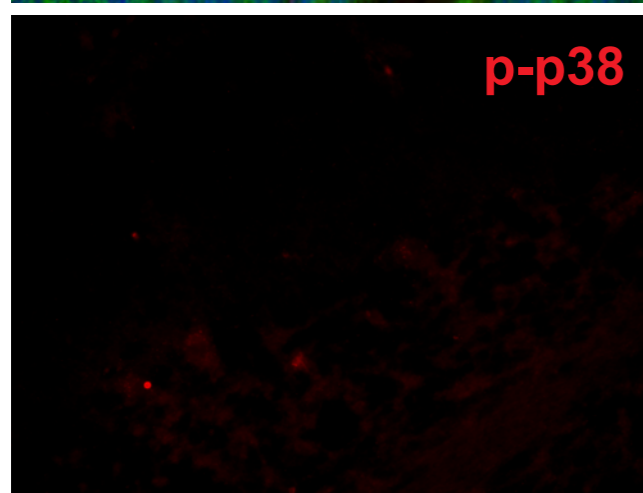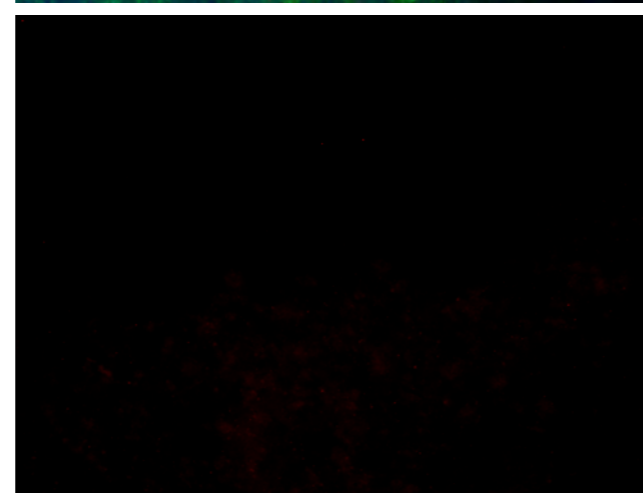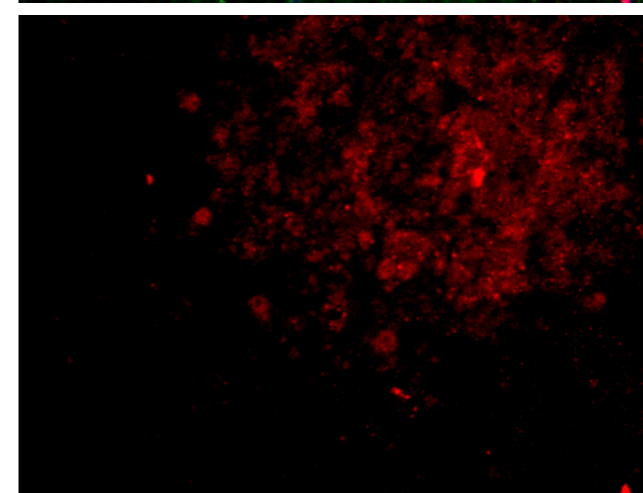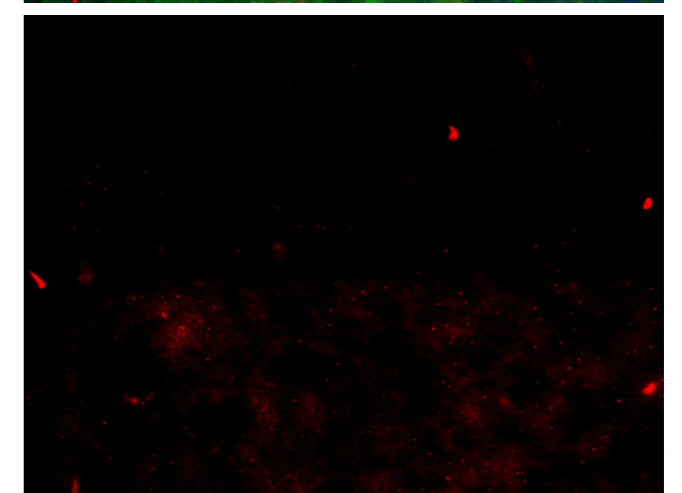

**Atm-/-**

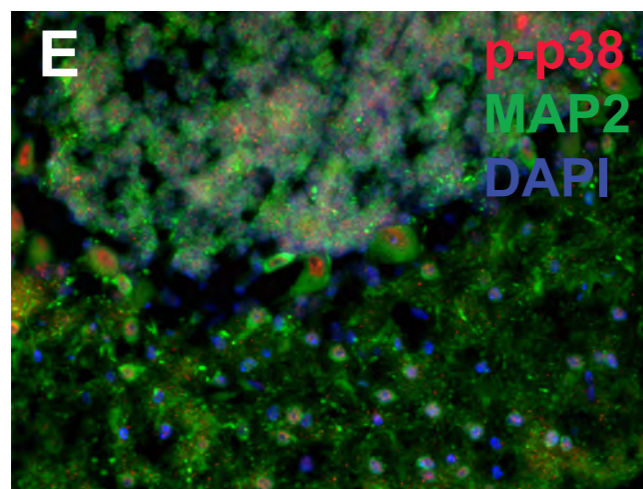

**Atm-/- (Ibu)**

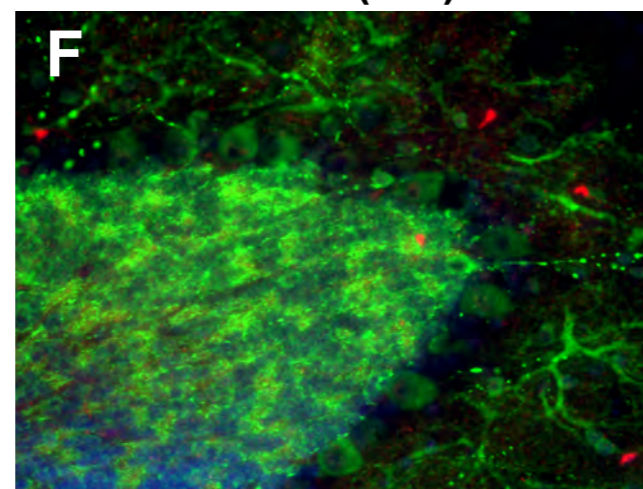

**Atm-/- (LPS)**

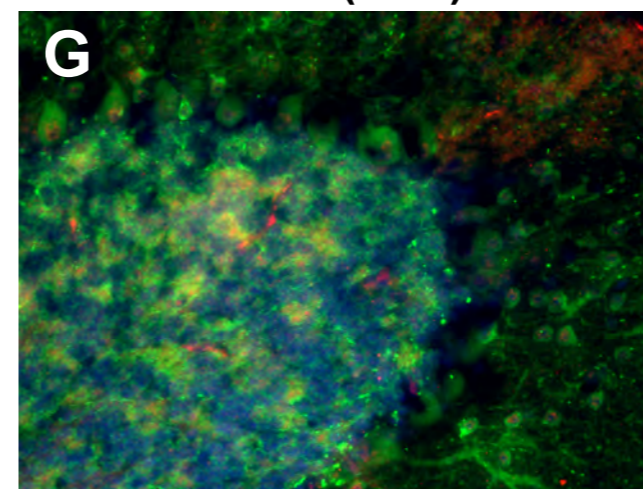

**Atm-/- (LPS+Ibu)**

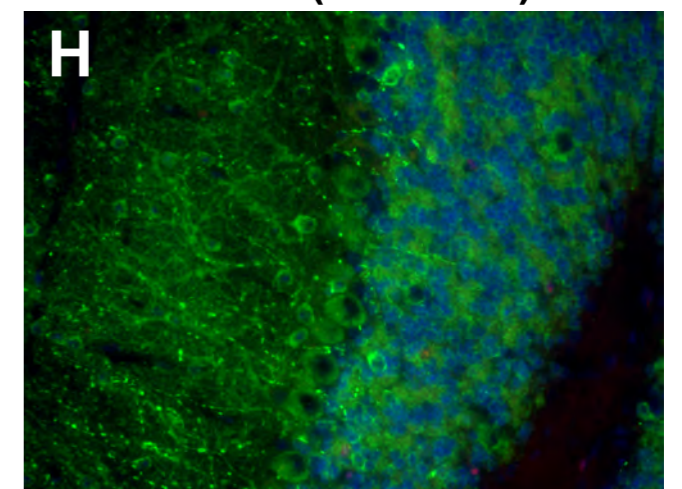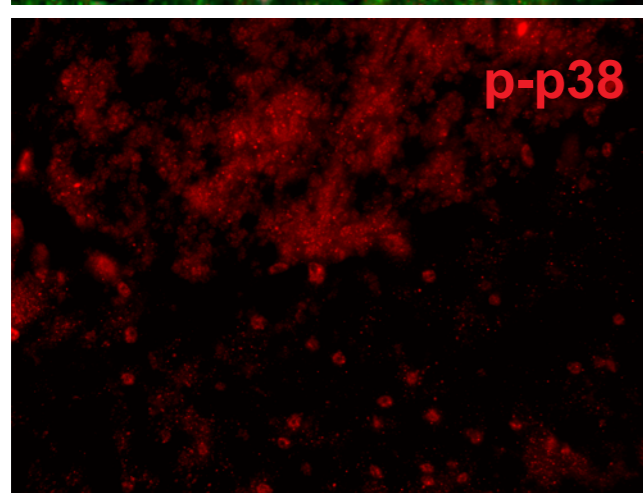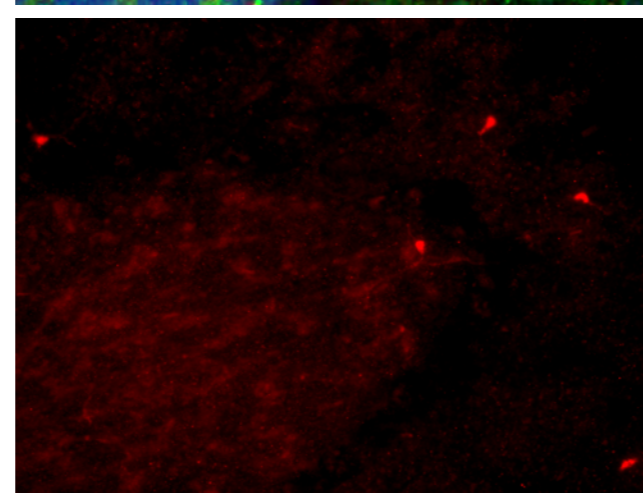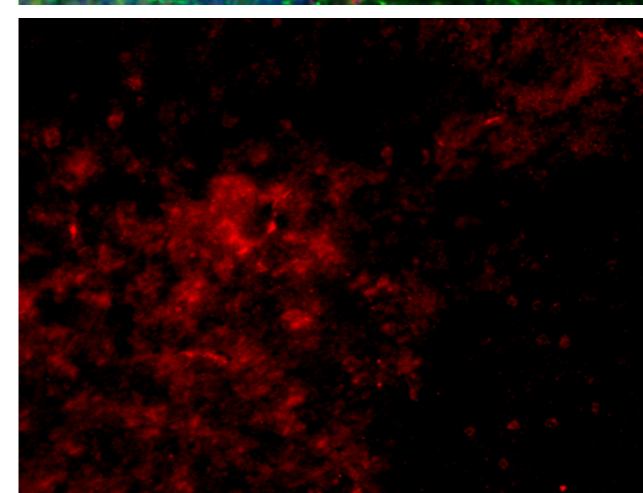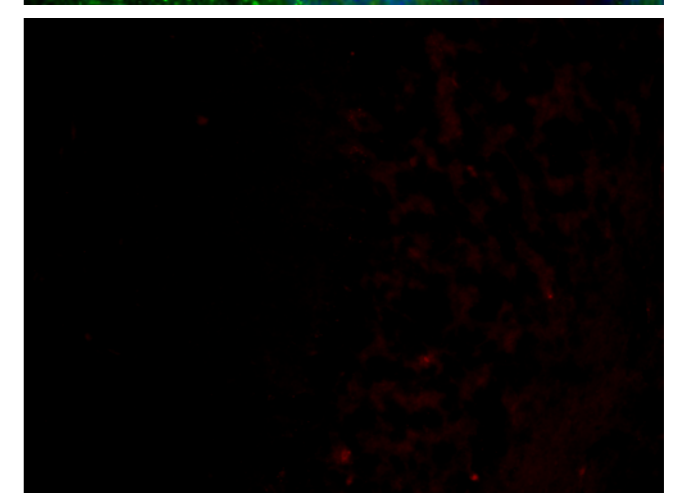

Supplement: Supplementary file 5 — Figure S5. Ibuprofen specifically suppresses p38 phosphorylation in PCs. Cerebellar sections were stained with phospho-p38 antiserum and visualized by fluorescent microscopy. LPS or ATM deficiency alone increased phospho-p38 levels in PCs (C, E) yet LPS unexpectedly reduced phospho-p38 levels in Atm−/− cerebellum (G). Ibuprofen treatment significantly reversed LPS triggered p38 phosphorylation in both Atm+/+ and Atm−/− PCs (D, H). (PDF 3474 kb) [file 12974_2018_1338_MOESM5_ESM.pdf]
